# The Ascent of Supramolecular Polymers and Gels in Asymmetric Catalysis

**DOI:** 10.1002/chem.202501446

**Published:** 2025-07-07

**Authors:** Ran Chen, Laurent Bouteiller, Matthieu Raynal

**Affiliations:** ^1^ Sorbonne Université, CNRS, Institut Parisien de Chimie Moléculaire 4 Place Jussieu Paris 75005 France

**Keywords:** asymmetric catalysis, chirality, supramolecular gels, supramolecular polymers, synthesis

## Abstract

Supramolecular polymers (SPs) and gels, formed by the spontaneous assembly of small molecules through various types of noncovalent interactions, are attractive materials for many applications. Their modularity also offers many opportunities in asymmetric catalysis that have been tackled in the last two decades and more intensively in the last one. In this review, strategies adopted to develop efficient asymmetric catalysts supported on SPs and gels are first categorized according to the chiral or achiral nature of the monomers used for their construction and second to their ability to be commuted into different states. Catalytic SPs have been described for which enantioselectivity stems mostly from the molecular chirality located next to the reactive group, or at opposite ends of the spectrum, exclusively from the chiral environment provided by the supramolecular helices. New paradigms revealed by these systems include (i) the organization of catalytic sites at the periphery of modular and well‐structured 1D assemblies, (ii) the possibility to conduct asymmetric reactions with a sub‐catalytic amount of chiral inducers and even in the absence of chiral monomers, and (iii) the development of a new class of switchable asymmetric catalysts.

## Graphical abstract

SPs and gels emerged as modular platforms for asymmetric catalysis. This review ranks literature reports according to the achiral or chiral nature of the monomers bearing the catalytic site. New paradigms showcased by this approach are particularly highlighted, such as catalysts operating with a low amount of chiral inducers, or with no chiral inducer, and the development of switchable asymmetric catalysts.

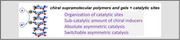

## 1 Introduction

The assembly of small molecules through noncovalent interactions generates supramolecular polymers (SPs), a class of material that possesses both polymer‐like properties such as viscoelasticity and unique properties such as stimuli‐responsiveness and self‐healing.^[^ ^1^ ^]^ Their potential in catalysis has been unlocked in the last two decades along with a variety of applications.^[^ ^2^, ^3^, ^4^, ^5^, ^6^, ^7^, ^8^ ^]^ Pioneering studies dealing with SP in catalysis focused on physical gels, which actually consist of bundled polymer chains, metastable fibers that formed upon cooling a solution of a low‐molecular‐weight compound. These gels, despite offering low diffusion kinetics for the reactants or catalysts that may be trapped, have been implemented as reaction vessels, reactive gels, and (less frequently) as catalysts, sometimes with interesting properties such as increased catalyst lifetime, enhanced activity, and unusual selectivity (all relative to the solution state).^[^ ^9^, ^10^, ^11^, ^12^ ^]^ Metallogels^[^ ^13^ ^]^ integrate metal atoms both as structuring elements and catalytic sites allowing recyclability.^[^ ^10^, ^14^ ^]^ Hydrogels, notably those formed by the assembly of peptides, have been widely investigated, mostly in the context of their ability to display enzyme‐like properties or trigger catalytic activity solely in the gel state.^[^ ^15^, ^16^, ^17^, ^18^ ^]^ It was soon realized that the fiber‐like morphology of the peptide aggregates present in these gels is beneficial for their hydrolytic activity.^[^ ^19^ ^]^ This was attributed to the higher internal order provided by the cylindrical nanofibers relative to spherical aggregates, which facilitates substrate binding. Small peptides able to assemble into fibrils,^[^ ^20^, ^21^ ^]^ nanotubes,^[^ ^22^ ^]^ and amyloid‐type aggregates^[^ ^23^, ^24^ ^]^ may exhibit remarkable properties such as enantiomer discrimination.^[^ ^24^ ^]^ However, the use of hydrogels as asymmetric catalysts remains relatively scarce; a few examples will be disclosed in the second part of this review.^[^ ^25^ ^]^ Dipeptides derived from histidine are potent asymmetric catalysts: it was soon suggested that their aggregation properties may be beneficial for this purpose^[^ ^26^ ^]^ even though rationalization of the catalytic outcome regarding the aggregation state could not be definitively ascertained.^[^ ^27^ ^]^ More recently, hydrogels embedding electron‐active chromophores coupled with suitable active metals were probed as catalysts for photocatalytic hydrogen production.^[^ ^28^, ^29^, ^30^ ^]^ In another approach, 1D coordination and organic polymers can be used to positions functional sites in adequate position in order to react smoothly and selectively in the solid or crystalline states.^[^ ^31^, ^32^, ^33^ ^]^

Single‐chain SPs formed in solution have been less investigated in catalysis than the aforementioned gels or coordination polymers that consist of crosslinked fibers or chains bundled to each other. It may seem a bit surprising at first sight, because diffusion is less hampered in these materials, but their limited stability may pose a unique challenge to sustain the catalytic conditions. SPs composed of achiral catalytically active monomers were found to be able to enhance the catalyst lifetime,^[^ ^34^ ^]^ and even the diastereoselectivity of a reaction.^[^ ^35^ ^]^ Implementation of SP in asymmetric catalysis is intimately related to the development of strategies to construct chiral SPs. Soon after the discovery that SP may form long assemblies in diluted solutions,^[^ ^36^ ^]^ the impact of chirality on their structure, composition, and stability has been a topic of intensive investigation.^[^ ^37^ ^]^

It was soon recognized that many of the intriguing chiral features exhibited by helical covalent polymers are also operative in chiral SPs.^[^ ^38^, ^39^ ^]^ A large number of SPs indeed adopt a helical configuration upon assembly or stacking of their constituting monomers.^[^ ^40^, ^41^, ^42^, ^43^, ^44^, ^45^ ^]^ The helical arrangement of the monomers at the supramolecular scale constitutes an additional element of chirality.^[^ ^46^ ^]^ Controlling the chirality at the supramolecular scale can be achieved “hierarchically” through chirality induction between the different partners involved in the construction of the SP. Employing chiral monomers appeared to be the most conventional way to control the main chain chirality of the SP, that is, its handedness and helicity, through a single chirality induction event between the stereogenic center of the chiral monomer and the main chain of the SP. The stereogenic center can be located at a reasonable distance from the SP main chain as long as the difference in energy between the *P* (right‐handed) and *M* (left‐handed) supramolecular helices is sufficiently high to promote the formation of a single‐handed SP. Similarly to antecedents in helical covalent polymers,^[^ ^38^ ^]^ it was uncovered that efficient control of the SP main chain chirality can be achieved even with a minute chiral perturbation of the environment of the SP. For example, even monomers with a single hydrogen atom substituted by deuterium in the methylene unit of its side chain, that is, isotopically chiral monomers, are able to induce a preferred handedness to the SP.^[^ ^47^ ^]^ This is due to the fact that a small chiral preference at the level of the repeating unit (as low as a few J.mol^−1^) creates a conformational bias that is sufficiently amplified through the whole nanostructure to lead to observable chiroptical properties at the macroscopic scale. Efficient induction of chirality is also achieved for SPs containing nonenantiomerically pure or achiral monomers. The chiral monomers, even though present in low amount or in moderate enantiomeric excess dictate, their chiral preference to the SP, eventually leading to homochiral SPs, that is, single‐handed SPs.^[^ ^40^ ^]^ For these supramolecular copolymers (SCPs), induction of chirality operates at several levels. When the chiral monomers (the “sergeants”) are able to control the helicity of an SCP composed of a large number of achiral monomers (the “soldiers”), the “sergeants‐and‐soldiers” (S&S) effect applies, whereas the majority‐rule (MR) principle operates when the induction of chirality goes through the presence of a major enantiomer of the chiral monomer. These two phenomena have been observed for a large number of SCPs: single‐handed SCPs have been obtained with less than 1% of “sergeants”,^[^ ^48^, ^49^, ^50^, ^51^, ^52^ ^]^ or with less than 10% enantiomeric excess.^[^ ^53^, ^54^ ^]^ Alternately, the rather weak chiral perturbation induced by chiral solvents,^[^ ^55^, ^56^ ^]^ or physical fields such as microvortices,^[^ ^57^, ^58^, ^59^ ^]^ circularly‐polarized light,^[^ ^60^, ^61^ ^]^ or chirality‐induced spin selectivity^[^ ^62^ ^]^ can be sufficient to afford efficient chirality induction in SP. In rare cases, single‐handed helical nanostructures often composed of very large fibers were generated in the absence of any chiral bias through spontaneous‐mirror symmetry breaking (SMSB) phenomena operating under far‐from‐equilibrium conditions.^[^ ^63^, ^64^, ^65^, ^66^ ^]^

**Figure 1:**
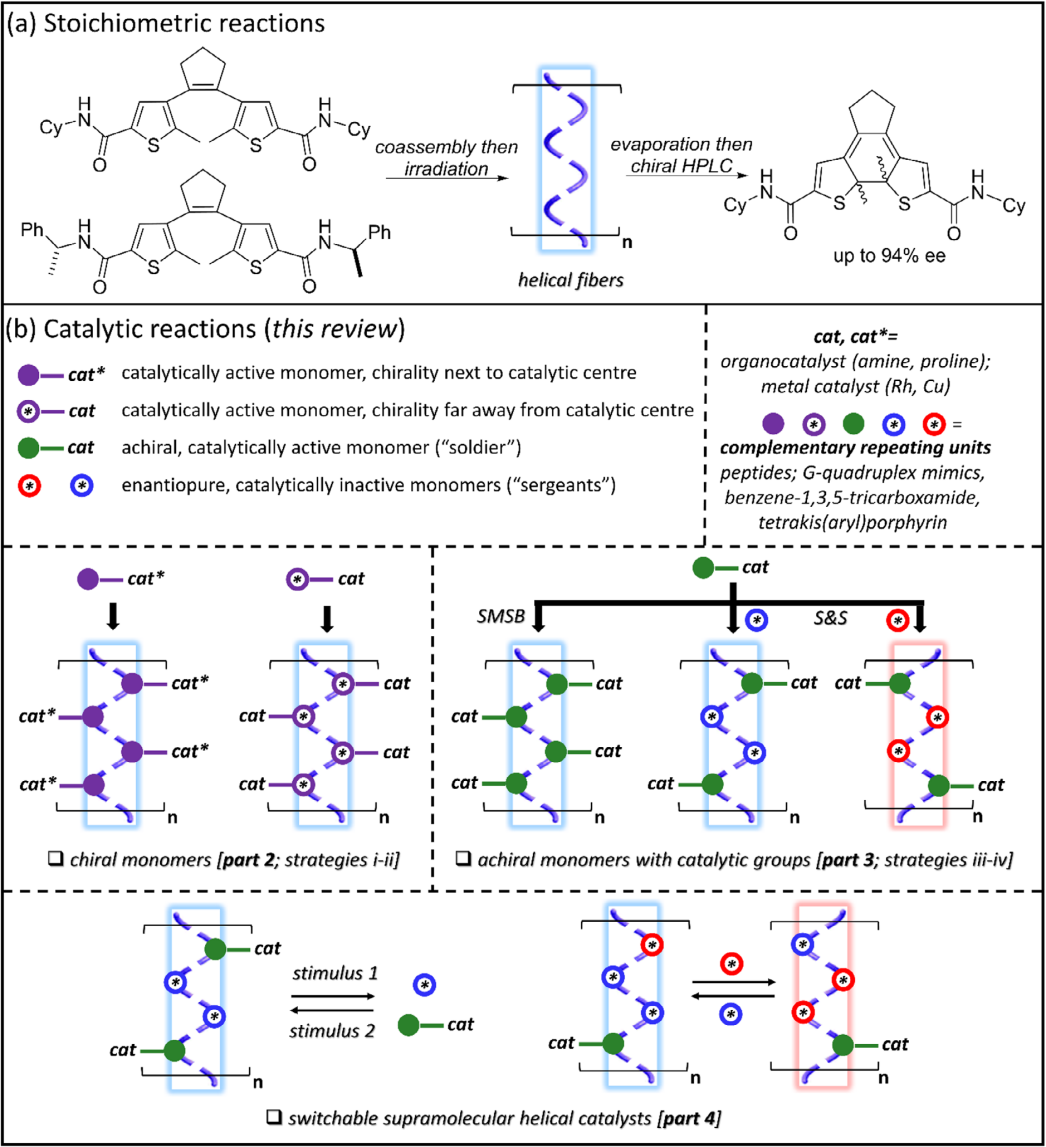
a) Example of an asymmetric reaction involving the constituting monomers of a supramolecular gel.^[^ ^63^ ^]^ b) Schematic representations of the four strategies used to construct catalytically active chiral SPs and SCPs. The main chain of the chiral SP corresponds to a helical arrangement of the monomers as represented as a dotted helix highlighted in blue and red for *P* (right‐handed) and *M* (left‐handed) helices, respectively. SMSB: SMSB. S&S: “S&S”.

Initial examples of reactivity in chiral SPs was reported in the early 2000s by the group of Feringa, who demonstrated that the photocyclization of dithienylethene‐based chiral monomers occurred with excellent diastereoselectivity solely when chiral monomers are aggregated, that is, that supramolecular chirality leads to induction of a preferential molecular chirality (Figure 1a).^[^ ^68^ ^]^ Selective cyclization was also achieved for SCP made from achiral and chiral dithienylethene‐based monomers, exploiting the aforementioned S&S effect.^[^ ^67^, ^69^ ^]^ The group of Shinkai observed similar phenomena in organogels of dimerizable anthracene‐based monomers.^[^ ^70^, ^71^ ^]^ The next step was to implement chiral SP and gels as a platform for catalysis. In order to make chiral SPs and gels capable of accelerating asymmetric reactions, well‐designed catalytic groups have been anchored to the monomers. To date, four main strategies have been implemented to construct catalytically active chiral SPs or gels: the stereogenic center and the reactive unit were located next to each other in the same monomer (strategy i); the stereogenic center and the reactive unit were located far away from each other in the same monomer (strategy ii); the reactive unit was located on an achiral monomer that experiences the chiral environment of the SP thanks to its homoassembly controlled by SMSB (strategy iii) or its coassembly with chiral monomers (strategy iv, S&S‐type helical catalysts). These four strategies are represented schematically in Figure 1b: the supramolecular gel or polymer catalysts are built with chiral monomers only (part 2, strategies i‐ii) or with achiral catalytically active monomers (part 3, strategies iii‐iv). To the best of our knowledge, the field of chiral gels and SPs in asymmetric catalysis has not been comprehensively reviewed, but selected examples have been mentioned in a few reviews dealing with supramolecular helical systems,^[^ ^39^ ^]^ chiral SPs,^[^ ^44^ ^]^ supramolecular chirality,^[^ ^42^ ^]^ self‐assembled chiral nanostructures,^[^ ^72^ ^]^ transmission of chirality,^[^ ^73^ ^]^ asymmetric amplification in catalysis,^[^ ^74^ ^]^ or the authors own work.^[^ ^75^ ^]^ The implementation of gels as reaction media to disperse nonassembled chiral catalysts is an alternative approach that is not covered in this review.^[^ ^76^, ^77^, ^78^ ^]^ It is worth noting that covalent helical polymers have been exploited almost conjointly in the context of asymmetric catalysis,^[^ ^74^, ^79^, ^80^, ^81^, ^82^ ^]^ with obvious differences in terms of structure, stability, and modularity relative to helical SPs but also similar features such as the development of S&S‐type,^[^ ^83^ ^]^ MR‐type^[^ ^84^ ^]^ and switchable helical catalysts.^[^ ^85^ ^]^ Here, efforts to develop SPs and gels in asymmetric catalysis made in the last two decades, and more intensively in the last one, will be ranked according to the aforementioned strategies, particularly emphasizing the role of supramolecular chirality in dictating the outcome of the catalytic reaction, that is, its influence on the enantioselectivity. It is indeed important to disentangle the contributions of the molecular and supramolecular chirality on the catalyst enantioselectivity in order to better apprehend these systems. Secondly, supramolecular helical catalysts that obey the S&S effect will be particularly stressed, as optimal selectivity in these systems does not strongly depend on the number of chiral monomers embedded into the SP. Finally, a family of switchable supramolecular helical catalysts for which the selectivity can be tuned will be presented in a dedicated part (part 4). The aim of this review is not to compare the catalytic systems reported to date but rather to delineate the strategies followed for their construction and to highlight their original features, notably in terms of chirality induction and commutation, in order to draw inspiration for future design and catalytic applications of these fascinating functional nanostructures.

## 2 Supramolecular Polymer and Gel Catalysts Made From Chiral Monomers

Chiral catalysts combining a catalytic active site for substrate activation and stereogenic elements in the same backbone to control the stereoselectivity are the most widespread class of asymmetric catalysts.^[^ ^86^ ^]^ While in most cases they promote the reaction as discrete species, assembly of chiral metal complexes and organocatalysts can also play a major role in dictating the outcome of a catalytic process, being at the origin of positive or negative nonlinear effects.^[^ ^87^ ^]^ Alternatively, assembled chiral catalysts may provide lower enantioselectivity than the discrete (nonassembled) ones, as exemplified by recent examples of hyperpositive nonlinear effects.^[^ ^88^ ^]^ The Soai reaction, an asymmetric auto‐catalytic reaction that yields an enantiopure product in the absence of detectable chiral species, also operates through complex assembled structures integrating the ligand(s), the metal center, and the reaction product.^[^ ^89^ ^]^ SPs constitute an attractive platform to control the spatial arrangement of catalytic centers and to tentatively rationalize their mode of action. The following part will be dedicated to supramolecular gels and polymers formed by assembly of enantiopure and catalytically active monomers, with a distinction between those bearing catalytic and stereogenic centers close to each other (part 2.1) and those for which the catalytic and stereogenic units are far away from each other (part 2.1).

### 2.1 Stereogenic Centre(s) Close to the Catalytic Site

#### 2.1.1 Organocatalytic Reactions

The discovery that amino acids^[^ ^90^, ^91^ ^]^ and most notably proline, were able to promote aldol and related reactions enantioselectively led to the development of organocatalysis as the prominent field of research that we know today. It was established from these seminal studies that the catalytic reaction is highly sensitive to the reaction medium, most probably because it may affect the hydrogen bonding interaction and the enamine configuration of the presumed Zimmerman‐Traxler‐type transition state established in the course of the aldol reaction.^[^ ^92^ ^]^ Proline has been anchored onto a large number of supports, notably dendrimers,^[^ ^93^ ^]^ and polymers.^[^ ^94^, ^95^, ^96^ ^]^ The corresponding hybrid systems have been tested in model reactions (often the aldol reaction) in order to reach several objectives, such as efficient catalysis in (or in the presence of) water, low catalytic loading, and/or recyclability. The 1D structure of supramolecular gels and polymers has been envisaged as an interesting approach to precisely organize proline moieties (and less frequently other organocatalytic groups) thanks to noncovalent interactions between well‐designed derivatives. Organogels, hydrogels, and chiral SPs bearing chiral organocatalytic groups will be treated successively in this subpart.

**Figure 2:**
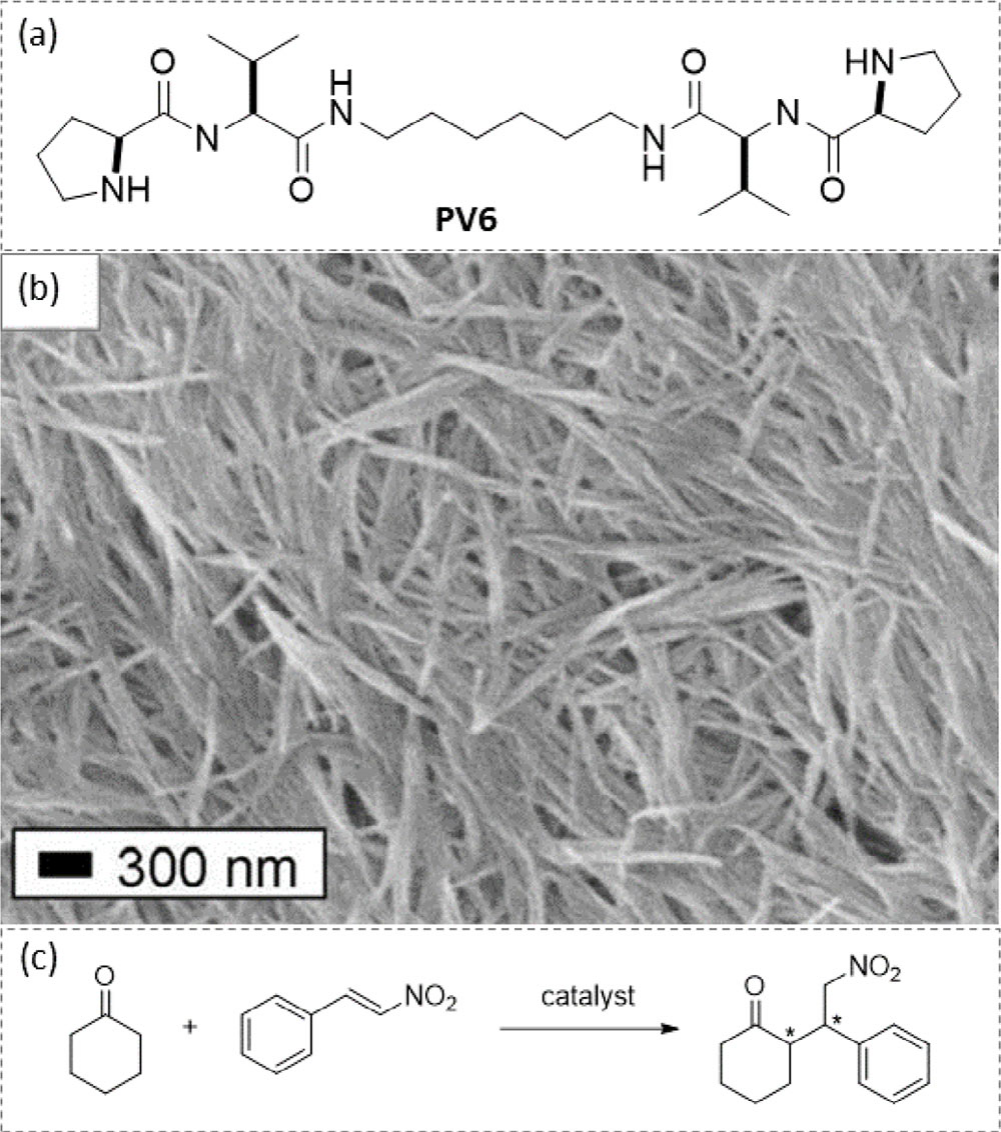
Chemical structure of **PV6** a), SEM image of a gel of **PV6** in toluene b), and reaction scheme of the 1,4‐conjugate addition c). The SEM image is reproduced from Ref. [98] with permission from the Royal Society of Chemistry.

In 2010, Escuder and Miravet reported on an L‐Proline derivative consisting of a pair of proline‐valine synthons connected by a hexamethylene linker (**PV6**, Figure 2).^[^ ^97^ ^]^ This bolaamphiphile forms an organogel in toluene; the presence of a self‐assembled fibrillar network (SAFiN) was confirmed by scanning electron microscopy (SEM, Figure 2).^[^ ^98^ ^]^ This network consists of large fibers that are usually more than 100 nm large and actually are bundles of many individual hydrogen‐bonded chains. No preferential helicity can be detected. The gel provided full conversion but modest enantioselectivity (34% enantiomeric excess, ee) in the 1,4‐conjugate addition of cyclohexanone to trans‐β‐nitrostyrene. Interestingly, model compounds in the nonaggregated state yielded the opposite enantiomer. This was attributed to the fact that the enamine formed by model compounds may engage in a hydrogen bond with trans‐β‐nitrostyrene in their corresponding transition state, while the amide groups of **PV6** are not available for substrate binding since they participate actively in the connection of the monomers in the aggregated state. A structurally simple proline‐tryptophan dipeptide was reported by the Liu group to form spheres and nanofibers in water and a dmso/water 1:2 mixture, respectively.^[^ ^99^ ^]^ However, it was found that the fibers present in the gel state provided low enantioselectivity in the aldol reaction between cyclohexanone and *p*‐nitrobenzaldehyde, conducted in the presence of water. This reaction is commonly used to assess the catalytic performance of newly‐developed organocatalysts, and it will be referred as the model aldol reaction in the following paragraphs. Catalytic results for this reaction for the main systems reported in part 2.1.1 are compiled in Table 1.

**Table 1:** Catalytic performance of proline‐derived supramolecular gels and polymers in the aldol reaction between cyclohexanone and *p*‐nitrobenzaldehyde.

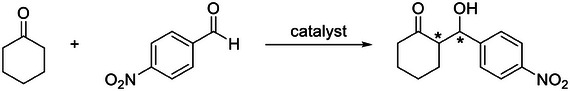					
Entry	Catalyst	yield [%]	dr [*anti*:*syn*]	ee [%]	Reference
1^[a]^	**L‐Pro‐L‐Glu**	95	13/1	95	[100]
2^[b]^	**PVC12**	99	12/1	88	[108]
3^[b]^	**G2**	89	4/1	91	[115]
4^[c]^	**L‐Pro‐NDI**	95	12/1	87	[119]
5^[c]^, ^[d]^	**BTA‐a**	99	11/1	99	[120]
6^[c]^, ^[d]^	**BTA‐c**	42	10/1	94	[120]
7^[c]^	**BTA‐d**	>99	12/1	94	[122]
8^[c]^	**BTA‐d **+ **BTA‐e**	92	21/1	97	[122]

^[a]^ Xerogel.

^[b]^ Hydrogel.

^[c]^ In water.

^[d]^ Temperature treatment (slow cooling, 20 K/h).

**Figure 3:**
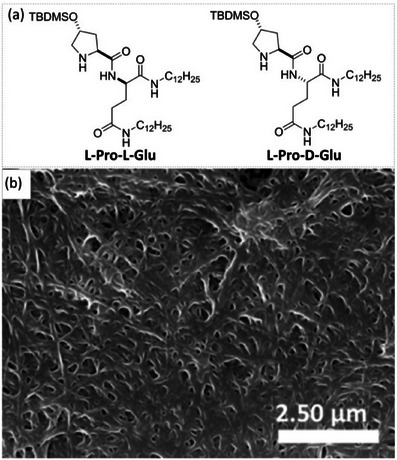
Chemical structures of **L‐Pro‐L‐Glu** and **L‐Pro‐D‐Glu** a) and SEM image of the **L‐Pro‐L‐Glu** xerogel formed from CH_3_CN/CHCl_3_ b). TBDM: tert‐butyldimethylsilyl. The SEM image is reproduced from Ref. [100], https://doi.org/10.1021/acsomega.8b00852, under the terms of the Creative Commons CC BY license, https://creativecommons.org/licenses/.

In a subsequent study, the Liu group investigated another dipeptide constructed on a different design: (*S*)‐4‐hydroxyproline protected by a tert‐butyldimethylsilyl group on the hydroxy function was coupled to amide‐functionalized‐*L* or‐*D* glutamic acid, yielding **L‐Pro‐L‐Glu** and **L‐Pro‐D‐Glu** monomers (Figure 3).^[^ ^100^ ^]^ Both stereoisomers form gels in a range of organic solvents, and the SAFiN consists of bundled fibers of about 0.1 − 0.3 µm width. However, **L‐Pro‐L‐Glu** showed better efficiency in forming supramolecular nanostructures, resulting in noticeable supramolecular chirality as detected by circular dichroism (CD) spectroscopy. Interestingly, the authors compared the efficiency in the model aldol reaction of these monomers under different forms: as solutions in water, as gels in organic solvents, and as xerogels (solids obtained after evaporation of the gels). All reactions were conducted in the presence of water with benzoic acid as a cocatalyst. The xerogel obtained from the acetonitrile/chloroform mixture provided the best performance (2 mol% loading): 95% yield, diastereomeric ratio (dr) of 93/7 (*anti*/*syn*), and 95% ee (Table 1, entry 1). The scope of the reaction was probed and appeared to be quite large since most of the tested aryl aldehydes and cyclic ketones yielded similar values, while lower enantioselectivities were found for acyclic ketones. While the exact mode of action of these heterogeneous systems is not well understood, it is important to notice that both **L‐Pro‐D‐Glu** and model compounds (used as nonassembled models of **L‐Pro‐L‐Glu**) furnished the aldol product with lower enantioselectivity than **L‐Pro‐L‐Glu**. It is thus reasonable to think that not only the stereogenic centre(s) close to the pyrrolidine unit of proline but also the supramolecular chiral arrangement provided by the aggregated monomers in the xerogel play a role in the efficient enantiodiscrimination process of **L‐Pro‐L‐Glu**. More recently, Zhang, Bai, and co‐workers reported that a *C*_2_‐symmetric hydroxyproline derivative efficiently promoted the aldol reaction between acetone and p‐nitrobenzaldehyde (up to 98% ee) and the Mannich reaction between the same components plus 4‐methoxyaniline (up to 99% ee) in water in its hydrogel form.^[^ ^101^ ^]^ The gel formed in water/dmso mixtures proved advantageous to improve the yield of the Mannich reaction as a consequence of the better dispersion of the reactants in this medium.

Mimicking the efficiency of aldolase^[^ ^102^ ^]^ and catalytic antibodies^[^ ^103^ ^]^ for the aldol reaction requires the preparation of proline derivatives since proline itself is not a competent catalyst for the reaction in water.^[^ ^92^ ^]^ Amphiphilic proline derivatives belonging to the family of surfactant‐type asymmetric organocatalysts^[^ ^104^ ^]^ are efficient catalysts for the Michael and aldol reactions in water. In this context, the hydrophobic environment provided by micelles,^[^ ^105^ ^]^ core‐shell polymeric micelles,^[^ ^106^ ^]^ vesicles,^[^ ^107^ ^]^ and single‐chain polymeric nanoparticles^[^ ^96^ ^]^ is a key factor to achieve high rates and selectivities. Supramolecular hydrogels^[^ ^17^, ^18^ ^]^ may operate through the same principle, thus offering an opportunity to finely tune the environment around the proline active center in the 1D aggregates.

**Figure 4:**
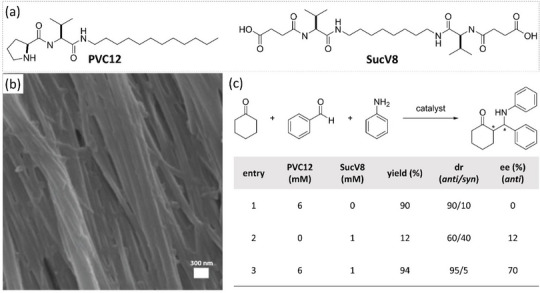
Chemical structures of **PVC12** and **SucV8** a) and SEM image of the **PVC12** hydrogel b). Catalytic performance of **PVC12**, **SucV8** and their mixture in the Mannich reaction c). The SEM image is reproduced from Ref. [108] with permission from the Royal Society of Chemistry.

In their seminal study in 2009,^[^ ^108^ ^]^ Miravet and Escuder conjointly reported a structurally simple catalytic amphiphile consisting of a proline‐valine head and an alkyl chain tail (**PVC12**, Figure 4a). This monomer self‐assembles in water to form a bilayer nanostructure, as evidenced by electron microscopy images and X‐ray diffraction (XRD) analyses. The SAFiN of the hydrogel is composed of very large fibers (Figure 4b). Catalytic experiments were conducted by adding the reactants in toluene on the top of the hydrogel. High conversion and selectivity were obtained for the model aldol reaction conducted at 5 °C (20 mol%): 99% yield, 92/8 (*anti*/*syn*) dr, and 88% ee (Table 1, entry 2). Remarkably, similar reaction efficiency and stereoselectivity were maintained after at least two additional runs, demonstrating the stability of the gel. In a subsequent study with the same hydrogel,^[^ ^109^ ^]^ a trend was observed between the yield in aldol product and the polarity of the ketone substrate: the ketone substrates with the longest alkyl pendants (2‐nonanone and 2‐dodecanone) provided the higher yields. The corresponding ee in aldol products was modest (30–40% ee), but only hydrogels proved to be active for these apolar substrates in water. This was attributed to the creation of a reaction compartment within the hydrogel that is able to accommodate apolar substrates thanks to the hydrophobic effect. Interestingly, it was found that **PVC12** and a bolaamphiphile with terminal acidic functions (**SucV8**, Figure 4a) self‐sort into their corresponding hydrogels. The isolation of the incompatible proline and acidic groups in their respective fibers makes it possible to perform a tandem deacetalisation/aldol reaction, affording the aldol product with an *anti*/*syn* ratio of 5.3 and 90% ee.^[^ ^110^ ^]^ **PVC12** hydrogel was also found to promote the Mannich reaction between aniline, benzaldehyde, and cyclohexanone, affording the *anti* stereoisomer of the β‐amino carbonyl product in good yield (Figure 4c).^[^ ^111^ ^]^ However, the reaction was not enantioselective (Figure 4c, entry 1). A positive influence on the enantioselectivity was found by mixing **SucV8** with **PVC12**: the *anti* product was now obtained with the same yield but 70% ee. This important effect is observed only if **SucV8** is added in its nongel form, such that it can be properly accommodated in the hydrophobic region provided by the hydrogel of **PVC12**, close enough to the proline unit to forge the cooperative activation of the substrates.

**Figure 5:**
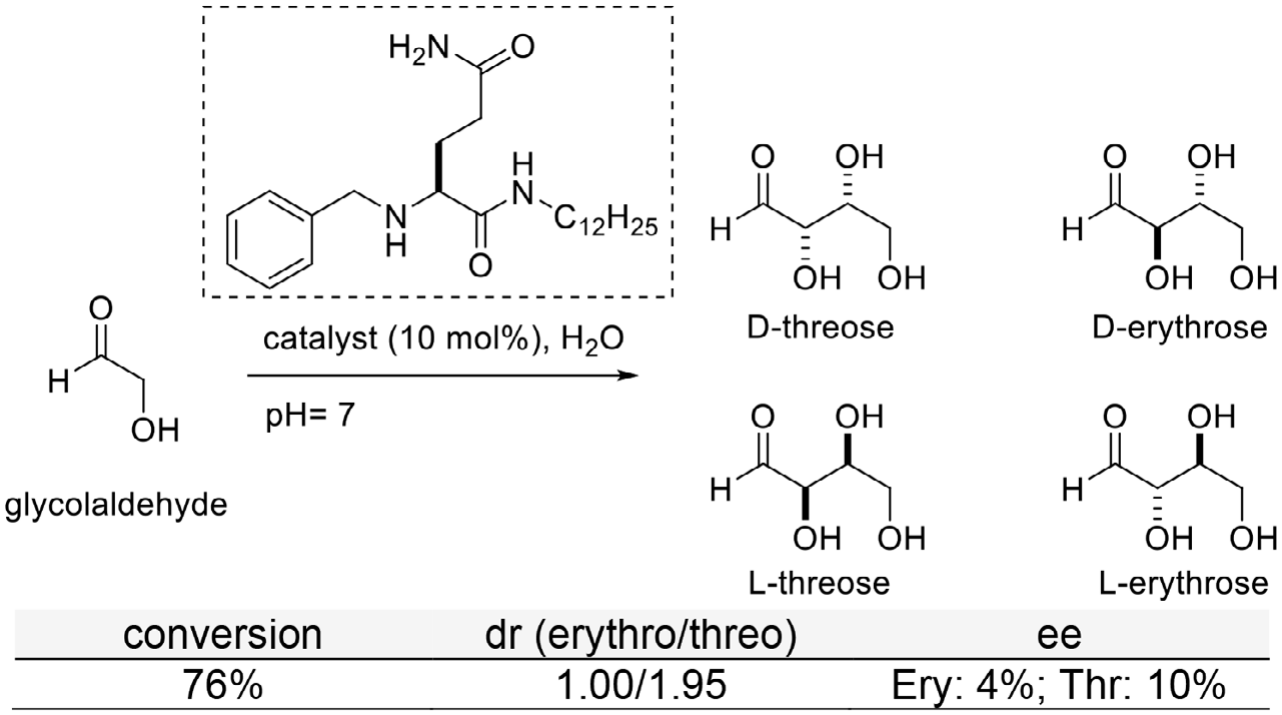
Self‐aldol reaction of glycolaldehyde catalyzed by a glutamine derivative.

A variety of tripeptides and decapeptides with terminal proline moieties were investigated with the aim of correlating the catalytic performance with the structures of the aggregates.^[^ ^112^, ^113^ ^]^ Tripeptide‐forming hydrogels proved to be the more potent catalysts, with the fastest rate being observed for the tripeptide having a phenylalanine moiety located next to proline as a probable result of its ability to bind more strongly to cyclohexanone.^[^ ^112^ ^]^ However, the reported enantioselectivity (66% ee) for the model aldol reaction is lower than the one provided by the aforementioned dipeptide **PVC12**. Supramolecular hydrogels were also tested for more challenging aldol reactions.^[^ ^112^, ^114^ ^]^ In this context, it is worth mentioning that the hydrogel resulting from the assembly of a structurally simple amphiphile derived from glutamine was found to promote the self‐aldol condensation of glycolaldehyde in convenient yields (76%), good stereoselectivity (threose:erythrose ratio of 2), but poor enantioselectivity (10% ee at best, Figure 5).^[^ ^114^ ^]^ Importantly, poor activity (5%) was detected for the same molecule in the solution state, thus highlighting the importance of the aggregation for catalysis. These results might be relevant to better apprehending prebiotic chemistry and the importance of simple amino acid derivatives as potential catalysts for the construction of polyol derivatives such as sugars.

**Figure 6:**
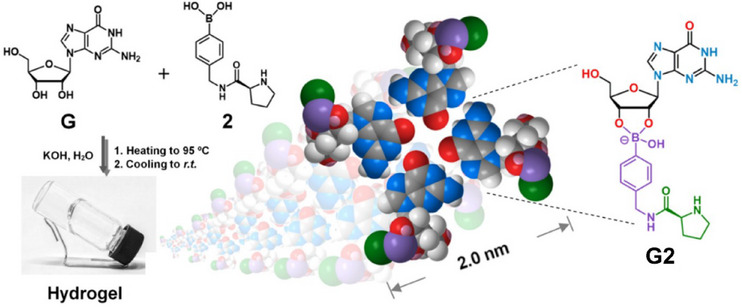
Two‐component hydrogel and schematic representation of the stacks formed by **G2**. Adapted from Ref. [115] with permission from the American Chemical Society.

Most of the supramolecular hydrogels investigated to date in asymmetric catalysis rely on peptides aggregation driven by hydrogen bonding, van der Waals and hydrophobic interactions. A different design was proposed by Qiao, Li, and coworkers.^[^ ^115^ ^]^ Inspired by the guanosine borate hydrogel reported by Davis et al.,^[^ ^116^ ^]^ these authors designed a two‐component hydrogel composed of guanosine and a boronic acid appended with a proline unit. In the presence of K^+^, a hydrogel consisting of thin fibers formed as a result of the stacking of **G2** reminiscent of the G‐quadruplex conformation adopted by guanine‐rich nucleic acid sequences (Figure 6). The hydrogel provided higher selectivity in the model aldol reaction (dr ratio of 4, 91% ee, Table 1, entry 3) than the individual components (46% ee at best). The assemblies formed in the presence of Na^+^ (not a hydrogel) were also significantly less selective, probably because their fibers are shorter than those formed in the presence of K^+^.

Supramolecular hydrogels are well‐established robust platforms to promote the hydrolysis of carboxylic esters. Schaaf, Jierry, and co‐workers developed an elegant system, for which assembly of peptides triggered by enzymes allows localization of the hydrogels on surfaces.^[^ ^117^ ^]^ Immobilization of the hydrogel formed by the enzyme‐assisted assembly of *Nap*‐GFFYGHY (*Nap *= naphthalene) in the cell of a commercial melamine foam enables the evaluation of its catalytic properties under continuous flow.^[^ ^118^ ^]^ The presence of histidine residues at proximal positions within the hydrogel is thought to promote the cooperative activation of carboxylic esters. The hydrogel is composed of helical fibers which are able to discriminate between the enantiomers of *tert*‐butyl esters derived from amino acids. Notably, the kinetic resolution of fmoc‐protected glutamic carboxylic ester enantiomers yields the corresponding acid in almost enantiopure form.

While the aforementioned hydrogels demonstrated their ability to promote asymmetric reactions, the rationalization of their catalytic outcome might be somewhat complicated because of their 3D network with multiple polymer chains connected to each other. Better‐defined supramolecular polymer assemblies have been investigated independently by the groups of Parquette and Meijer.

**Figure 7:**
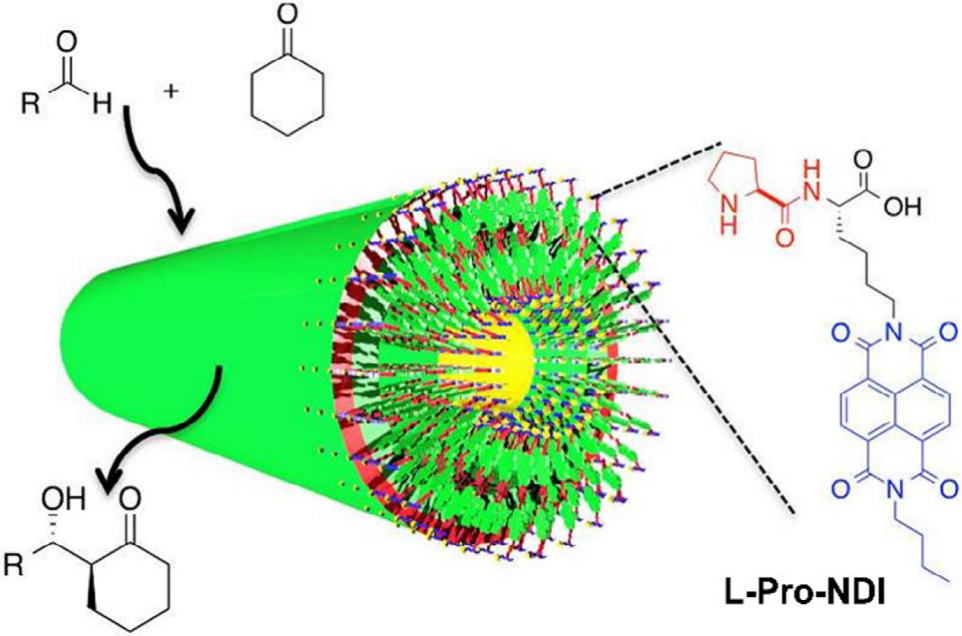
Chemical structure of **L‐Pro‐NDI** and schematic representation of its assembly into a supramolecular nanotube. Adapted from Ref. [119] with permission from the Royal Society of Chemistry.

In 2015, Parquette and coworkers designed an amphiphilic **L‐Pro‐L‐Lys** dipeptide analogue for which the lysine was functionalized with an NDI moiety (Figure 7 and L**‐Pro‐NDI**, NDI = 1,4,5,8‐naphthalenetetracarboxylic acid diimide).^[^ ^119^ ^]^ Atomic Force Microscopy (AFM) and Transmission Electron Microscopy (TEM) images of the assemblies of **L‐Pro‐NDI** in water in the presence of the substrates of the model aldol reaction (pa‐nitrobenzaldehyde and cyclohexanone) reveal isolated nanotubes for which the diameter of the walls is consistent with a bilayer arrangement of **L‐Pro‐NDI** (see a schematic representation in Figure 7). The proline residues are positioned both along the inner and outer surfaces of the nanotubular structure. The aldol reaction is slow (120 hours at 2.5 mol% loading) but occurs with good levels of dia‐ and enantioselectivity (*anti*:*syn *≈ 13, 87% ee, Table 1, entry 4). The system still exhibits some activity at low catalytic loading (0.5 mol%), conditions under which a nonassembled proline, *N*‐benzyl prolinamide, is not active. The addition of 2,2,2‐trifluoroethanol (TFE) to the catalytic mixture caused the nanotube to disassemble into monomeric dipeptides, which showed diminished activity but preserved enantioselectivity toward the catalytic aldol reaction. This indicates that (i) the hydrophobic region of the nanotubes is key to promote the reaction and (ii) even though the nanotube adopts a helical arrangement (as confirmed by CD analysis), the selectivity stems primarily from the L‐proline residue.

**Figure 8:**
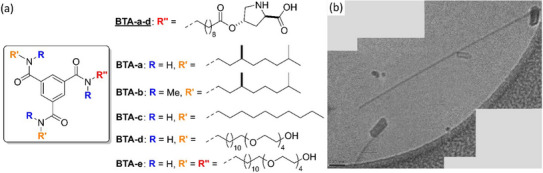
Chemical structures of the BTA monomers investigated in the model aldol reaction a). Cryo‐TEM images of **BTA‐a** (5 × 10^─4^ M) in water after temperature treatment; the scale bar represents 100 nm (bottom left, b). Reproduced from Ref. [120] with permission from Wiley‐VCH Verlag GmbH & Co. KGaA, Weinheim.

In 2015, Meijer, Palmans, and coworkers incorporated chiral proline moieties into rod‐like helical SPs based on benzene‐1,3,5‐tricarboxamide (BTA, Figure 8).^[^ ^120^ ^]^ These monomers are well‐established synthons in supramolecular chemistry, given their ability to predictably assemble into well‐defined supramolecular helices stabilized by triple hydrogen bonding and aromatic interactions under a large variety of conditions.^[^ ^121^ ^]^ In their initial design (monomers **BTA‐a**, **BTA‐b,** and **BTA‐c** in Figure 8a), the side chains of the *C*_2_‐symmetrical BTA monomers have been selected as follows: (i) two amide are connected by the same alkyl group, which can be achiral or chiral (3,7‐dimethyloctyl), (ii) one amide arm consists of a long alkyl tail terminated by a hydroxyproline unit through an ester linkage. Proline‐functionalized hydrogen‐bonded assemblies formed even in the presence of the substrates. However, formation of the assemblies was not spontaneous and required either ultrasonic or thermal treatment. Remarkably, the reactivity and selectivity for the model aldol reaction were significantly increased when the supramolecular catalyst composed of **BTA‐a** was subjected to a temperature treatment (heating to 343 K and then slow cooling to room temperature) compared to catalysis without temperature treatment. A 21‐fold improvement in the turnover frequency (TOF) was indeed determined for a catalyst loading of 1 mol%. This was attributed to a better packing of the proline unit, facilitating substrate binding to the supramolecular polymer, thus providing the aldol product with good selectivity (*anti*:*syn *= 9:1, 99% ee, Table 1, entry 5). Cryo‐TEM images of assemblies of **BTA‐a** after thermal treatment (Figure 8b) reveal aggregates with an average diameter of 80 nm, thus constituted of individual chains (2 nm) bundled together, and lengths varying between 300 and 600 nm. The impact of both the aggregation and supramolecular chirality of the aggregates on the catalytic outcome was precisely probed by studying structural analogues of **BTA‐a**. A BTA monomer with methylated amide functions, **BTA‐b**, unable to stack through hydrogen bonds, is as selective as **BTA‐a** but far less active. In addition, a BTA proline monomer with achiral side chains, **BTA‐c**, is significantly less active but only slightly less selective (Table 1, entry 6). These control experiments demonstrate that while the selectivity of the reaction is mainly dominated by the isolated chiral proline residues, the activity of the reaction strongly depends on the packing of the proline units in the supramolecular polymer.

In a subsequent work,^[^ ^122^ ^]^ the two alkyl side chains were changed to tetra(ethylene oxide) dodecyl groups to enhance water compatibility while keeping the third arm identical to that of **BTA‐a** (**BTA‐d** in Figure 8a). The dodecyl groups were introduced between the central amide groups and the peripheral water‐soluble parts to facilitate hydrogen‐bonding interactions between the amides of neighboring BTAs. CD, small‐angle X‐ray scattering (SAXS), and cryo‐TEM analyses of assemblies of **BTA‐d** highlight the formation of 1D, helical SPs in water with a biased helicity even though the proline unit is located far away from the BTA core. The observed fibers are thinner (≈ 10 nm) than those formed by **BTA‐a** as a probable consequence of the minimization of polymer chain bundling thanks to the peripheral hydrophilic groups. These assemblies demonstrated excellent activity and selectivity in the model aldol reaction with 1 mol% catalyst loading (*ant*i:*syn *= 12, 94% ee, Table 1, entry 7). Additionally, introducing 50% of the proline‐free BTA co‐monomer (**BTA‐e** in Figure 8a) into the assemblies did not erode activity and even increased the selectivity (*anti*:*syn *= 21, 97% ee, Table 1, entry 8). Mixing BTA monomers constitutes an easy way to modulate the local density of L‐proline units on the supramolecular polymer as well as the microenvironment around these units. This interesting strategy appears quite general for the optimization of supramolecular polymer catalysts by screening various combinations of co‐monomers.

#### 2.1.2 Metal‐catalyzed Reactions

Metal atoms can easily be integrated into organic‐based SPs either as a structural element to trigger the assembly, such as in metallo‐supramolecular or supramolecular metal‐coordination polymers, or as an additive to tune their properties, or both. Catalysis is an obvious application of metal‐organic SP hybrids on the condition that the metal contains free coordination sites available for substrate binding and activation. In the realm of asymmetric catalysis, the main challenges are of two kinds: (i) preserving the assemblies during the whole process, including the multiple catalytic steps, and (ii) providing a chiral environment that can efficiently discriminate the diastereoisomeric intermediates. Metal‐containing gels and SPs, with stereogenic centers close to the metal centers, have been applied in asymmetric catalysis; these examples are described below.

**Figure 9:**
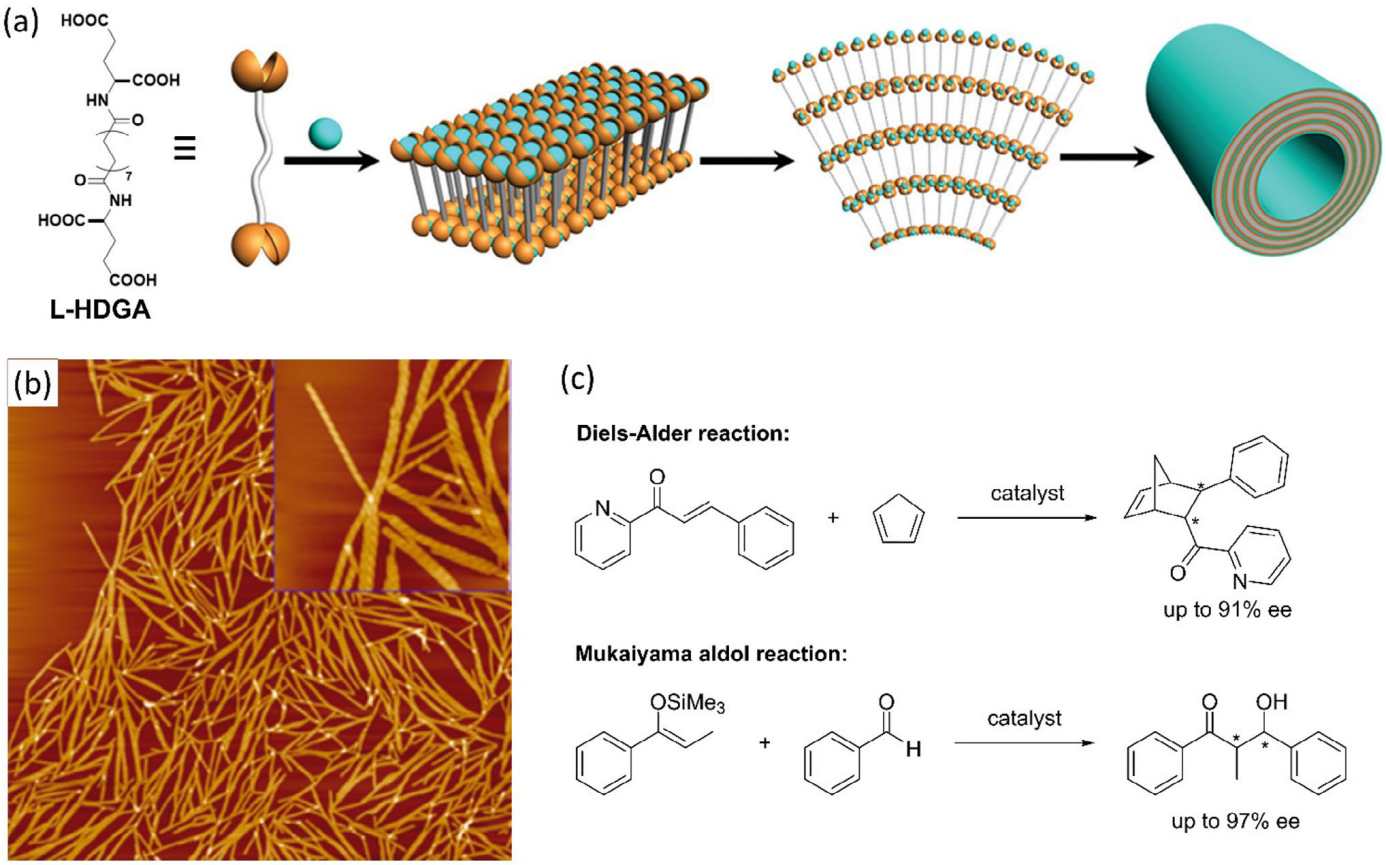
Chemical structure of **L‐HDGA** and schematic representation of its assembly into multi‐walled nanotubes a). Adapted from Ref. [124] with permission from the American Chemical Society. b) AFM images of **L‐HDGA** nanotubes loaded with Bi(OTf)_3_ (Bi:**L‐HDGA** ratio of 1/50). The size of the image is 5 × 5 µm^2^; the size of the enlarged part is 1 × 1 µm^2^. Reproduced from Ref. [125] with permission from the American Chemical Society. c) Reactions catalyzed by **L‐HDGA** metal hybrids with the highest observed selectivities.

Liu and coworkers reported that a bolaamphiphile derived from glutamic acid (*N*,*N*’‐hexadecanedioyl‐di‐L‐glutamic acid, **L‐HDGA**, Figure 9a) assembles into long helical nanotubes forming a hydrogel.^[^ ^123^ ^]^ In 2011,^[^ ^124^ ^]^ copper(II) ions were introduced as the catalytic centers thanks to their coordination to well‐arranged carboxylates located on the surfaces of the nanotubes, which was verified by a series of characterization techniques. A structural transition was observed from monolayer to multilayer nanotubes upon anchoring of the copper(II) ions onto the nanotubes. Pre‐formed chiral copper nanotubes, dispersed into water, promoted the asymmetric Diels‐Alder cycloaddition between azachalcone and cyclopentadiene with up to 97/3 dr and 51% ee for the major *endo* stereoisomer. Importantly, control catalytic experiments with pristine L‐glutamic acid, its *N*‐Boc derivative, and an amphiphile with a single head L‐glutamic acid as catalysts provided the *endo* product with less than 8% ee. **L‐HDGA** in its monomeric state (in ethanol) or in a nontubular assembly state (in a basic aqueous phase) also catalyzed the reaction but without enantioselectivity. These results nicely demonstrate that the enantioselectivity of the reaction mostly stems from the supramolecular chirality of the nanotubes, not from the molecular chirality of the individual monomers, even though the stereogenic centers are close to the copper active sites.

The multilayer structure of the bolaamphiphile‐based nanotubular catalyst prevents part of the added Cu^2+^ centers from being exposed to the substrates due to their intercalation in between the layers. In 2016, the same group managed to fabricate single‐walled nanotubes, again based on the self‐assembly of **L‐HDGA**.^[^ ^125^ ^]^ In this case, **L‐HDGA** monomers were first self‐assembled in water and subsequently dispersed into an aqueous solution containing the metal ions. This two‐step protocol enables a precise tuning of the nature and amount of metal centers relative to **L‐HDGA** (Figure 9b). Single‐walled nanotubes were found to be the main species when 0.1–2 mol% of metal ion was added to **L‐HDGA,** while higher ratios led to aggregation or collapse of the nanotubular structures. The asymmetric Diels‐Alder cycloaddition between cyclopentadiene and azachalcone was then reinvestigated with the single‐walled helical nanotubes Cu(II)‐HN (HN stands for helical nanotubes). Up to 99% yield, 92/8 dr, and 91% ee were reached with 0.1 mol% Cu^2+^ loading within 60 minutes, which is much more efficient and selective than the aforementioned multi‐walled helical nanotubular catalysts (Figure 9c). Variation on the azachalcone structures was well‐tolerated, since excellent yields and dr were obtained for five other candidates with good enantioselectivities (75%–90% ee). As anticipated the Cu:**L‐HDGA** ratio proved to be crucial, the highest enantioselectivity being obtained with a ratio of 1/100 probably because, at a higher ratio, the excess ions would not be controlled by the nanotube chirality. Control experiments further support the fact that the supramolecular nanotubular structure improve both the enantioselectivity and reactivity of the catalytic reaction. The modularity of the assembly process motivated the authors to probe the influence of different metal ions on a model Mukaiyama aldol reaction (Cu^2+^, Zn^2+^, Fe^2+^, Yb^3+^, Sc^3+^, Eu^3+^, Gd^3+^, Bi^3+^, and Pr^3+^). Interestingly, bismuth hybrid nanotubes (Bi(III)‐HN) with a Bi: **L‐HDGA** ratio of 1/50 outperformed all other metal nanotubes in the reaction between the silyl enol ether of propiophenone and benzaldehyde (Figure 9c). Through a comprehensive conditions screening, 91% yield, 94/4 *syn*/*anti* ratio, and 94% ee could be reached. Other (hetero)arylaldehydes were well tolerated (6 examples, 69–97% ee) but not (cyclo)alkyl ones. For both the Diels‐Alder and Mukaiyama aldol reactions, the metal ions were suggested to act as Lewis acids through binding and activation of azachalcone or aldehyde substrates. This was supported by the observation of an induced CD signal for the azachalcone substrate when bound to the copper hybrid nanotube. The curved surface of the supramolecular nanotubular structures is thus thought to align the bound substrate(s) in a helical way, thus allowing the second reactant to add selectively on one of its two prochiral faces.

**Figure 10:**
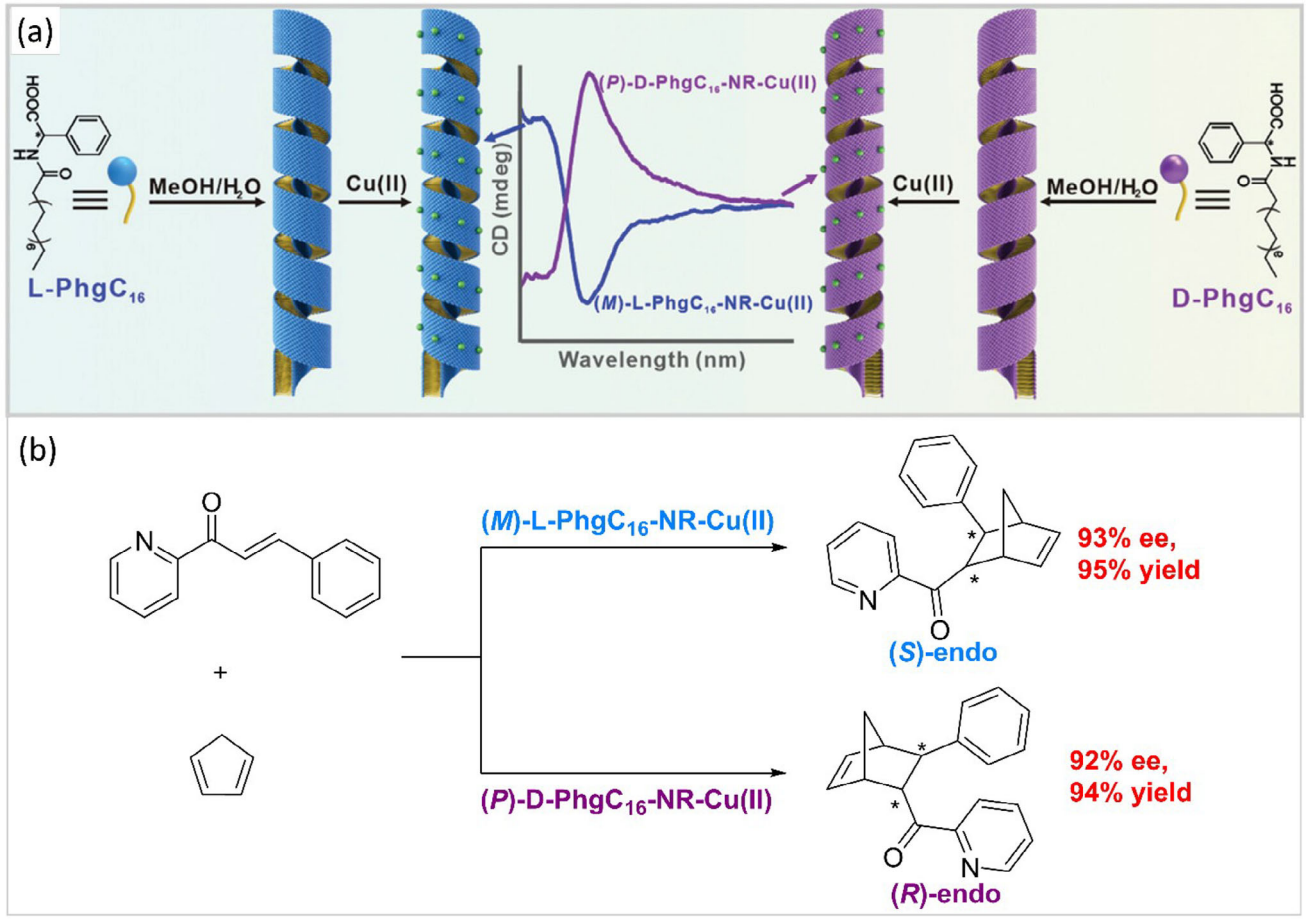
a) Chemical structure of **L‐PhgC_16_** and **D‐PhgC_16_** enantiomers and schematic representation of their assembly into helical nanoribbons. CD spectra of the assemblies formed in a 4/6 methanol/water solvent mixture. b) Catalytic performance of the copper hybrid nanoribbons in the Diels‐Alder reaction. Reproduced from Ref. [126] with permission from Wiley‐VCH Verlag GmbH & Co. KGaA, Weinheim.

Taking inspiration from the aforementioned work, Guo, Han, and coworkers reported in 2023 that simple single‐head amphiphiles with a terminal acidic function, **PhgC_16_** (Figure 10a), behaved similarly to **L‐HDGA** in promoting the asymmetric copper‐catalyzed Diels‐Alder reaction between azachalcone derivatives and cyclopentadiene.^[^ ^126^ ^]^ In that case, the pristine amphiphiles formed nanoribbons in the MeOH/water mixture, the structure of which was not significantly impacted by the coordination of the copper atoms. AFM images of the copper‐coordinated self‐assemblies revealed left‐ and right‐handed helical nanoribbons for **L‐PhgC_16_‐NR‐Cu(II)** and **D‐PhgC_16_‐NR‐Cu(II)**, respectively (see a schematic representation in Figure 10a, NR stands for nanoribbons). Unlike **L‐HDGA**, the mode of preparation of the nanoribbons did not influence their structure; that is, pre‐formation of the amphiphile assemblies prior to coordination to copper was not required. Optimizing the MeOH/water ratio and the Cu^2+^ catalytic loading yielded the Diels‐Alder product in good yield and selectivity (Figure 10b, 12 examples, 90–96% yield, 95:5–98:2 *endo*/*exo*, 71–96% ee). Screening of various amino acid derivatives revealed that **PhgC_16_** is the best‐performing one, even compared to those that assembled into helical nanotubes.^[^ ^127^ ^]^ Control experiments confirmed that, similarly to **L‐HDGA**, the enantioselectivity comes primarily from the helical arrangement of the amphiphiles in the nanoribbons. The glutamic acid analogue of **PhgC_16_**, **GluC_16_**, also forms helical nanoribbons, for which the screw pitch distance can be modulated by the choice of the organic cosolvent used in the assembly process.^[^ ^128^ ^]^ Interestingly, nanoribbons with the lowest pitch distance provided the highest ee; this was attributed to stronger interactions and better chirality transfer between the substrates and the nanoribbons.

**Figure 11:**
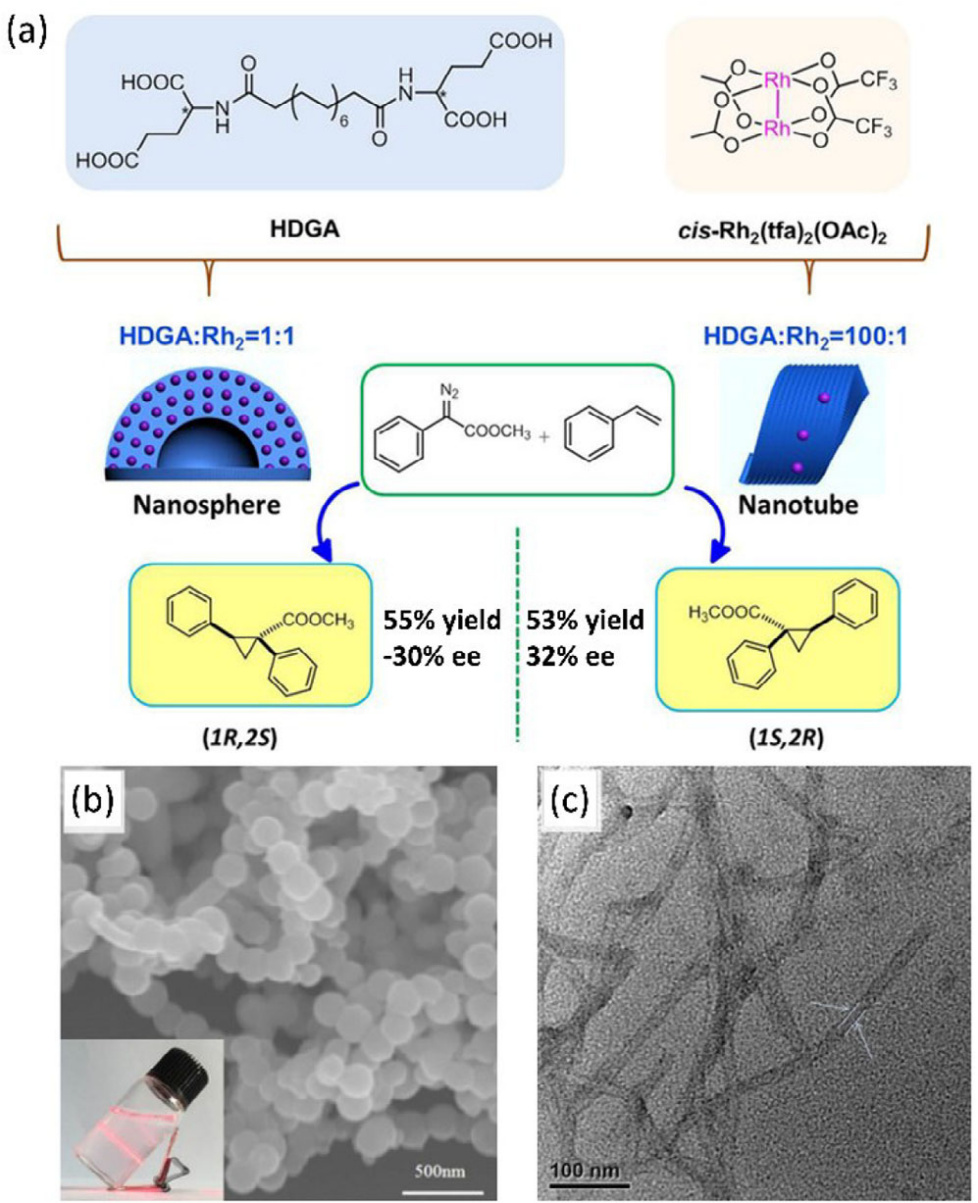
a) Schematic representation of the assemblies formed between **HDGA** and the Rh(II) precursor. Best catalytic performance of the nanosphere and nanotube assemblies in the rhodium‐catalyzed cyclopropanation reaction. b) SEM image of the assembly formed by mixing equimolar amounts of the Rh(II) precursor and **HDGA**. Inset: photograph of the assemblies formed in water (coloration is due to the Tyndall effect). c) AFM image of the assembly formed with a Rh(II) precursor/**HDGA** ratio of 1/100. Reproduced from Ref. [134] with permission from Wiley‐VCH Verlag GmbH & Co. KGaA, Weinheim. Copyright 2018.

While the aforementioned results employed the metal centers exclusively as Lewis acids,^[^ ^129^, ^130^, ^131^, ^132^, ^133^ ^]^ it is of interest to see whether these amino‐acid‐based metal chiral nanoassemblies could sustain other activation modes. Liu and coworkers investigated the self‐assembly behavior of **L‐HDGA** in the presence of a rhodium (II) dimer Rh_2_(tfa)_2_(OAc)_2_ (abbreviated as Rh_2_; tfa = trifluoroacetate, Figure 11a).^[^ ^134^ ^]^ It is disclosed that when the Rh_2_/**L‐HDGA** ratio was set to 1/100, a helical nanotubular structure formed which is similar to the ones described above for other metal ions. Fourier‐Transform Infrared (FT‐IR), XRD, and electron microscopy analyses verified that the formed helical nanotubes were nearly identical to those assembled from pure **L‐HDGA** (Figure 11c). Remarkably, when the Rh_2_ precursor was added to more diluted solutions of **L‐HDGA** and with an Rh_2_/**L‐HDGA** ratio of 1, the structure of the assembly turned out to be a hollow nanosphere, which was unambiguously demonstrated by SEM images (Figure 11b). Surprisingly, the CD signals induced to the acid‐coordinated Rh_2_ species have inversed signs for the nanospheres and nanotubes, which indicates that the Rh^2+^ centers in these nanoassemblies experience opposite chiral environments. The catalytic performances of the nanotube and nanosphere assemblies were further investigated for the asymmetric cyclopropanation model reaction between methyl phenyl diazoacetate and styrene, an organometallic reaction that involves a reactive rhodium carbene intermediate (Figure 11a). The nanotube catalyst provided the product in 53% yield and 32% ee, while the nanosphere catalyst yielded the other enantiomer of the product as the major enantiomer of the catalytic reaction (─30% ee). Again, disrupting the assemblies by the addition of NaOH gave the product in a racemic form. Different enantiomers of the product were thus favored by means of supramolecular chiral nanostructures originating from the same chiral monomer, unambiguously revealing the role of supramolecular chirality in dictating the selectivity outcome of the catalytic reaction.

### 2.2 Stereogenic Centre(s) Far From the Catalytic Site

**Figure 12:**
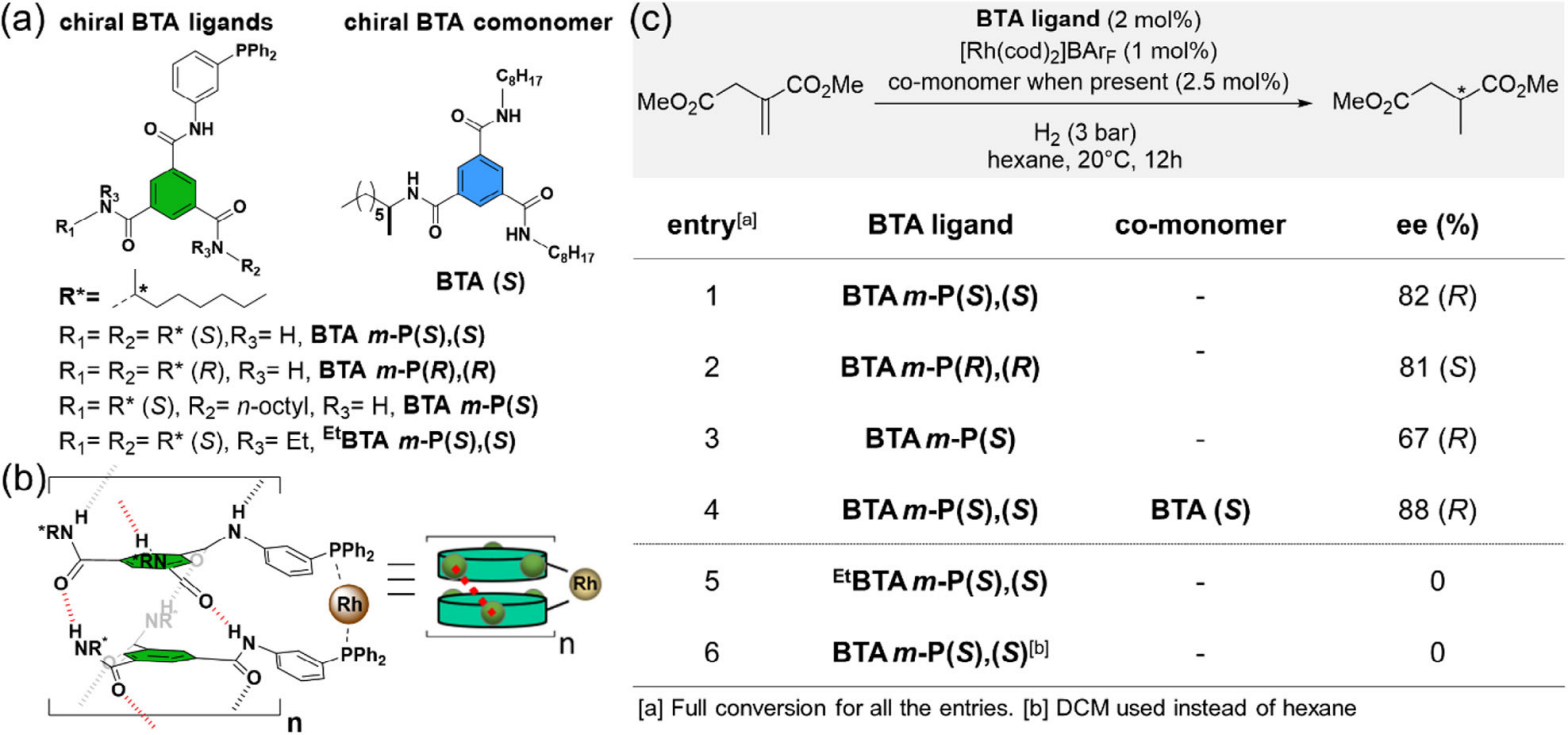
a) Chemical structures of the chiral BTA ligands and of the chiral BTA co‐monomer. b) Schematic representation of the chiral SP formed by **BTA *m*‐P(*S*),(*S*)** coordinated to rhodium. c) Catalytic performance of the BTA ligands in the rhodium‐catalyzed hydrogenation reaction.

The presence of a chiral center close to the catalytic site may lead to conflictual influence of the molecular and supramolecular chiralities on dictating the stereochemical outcome of an asymmetric reaction triggered by a chiral SP. In addition, the contribution of supramolecular chirality to the enantioselectivity was clearly established only for a few of the aforementioned examples. In 2013, our group probed the possibility of inducing enantioselectivity with chiral SP embedding chiral monomers for which the stereogenic centers were located far from the catalytic site.^[^ ^135^ ^]^ We designed and synthesized a series of chiral BTA‐based ligands that contained the same *m*‐phenylene diphenylphosphino group but differed by the nature of the alkyl side chains on the two remaining amide functions (either chiral 1‐methylheptyl or achiral n‐octyl, Figure 12a). The supramolecular helical rodlike structure of the self‐assembled monomer, **BTA *m*‐P(*S*),(*S*)**, was confirmed by FT‐IR and small‐angle neutron scattering (SANS) analyses, which were in agreement with literature data on nonfunctionalized BTA derivatives (Figure 12b). Upon coordination of [Rh(cod)_2_]BAr_F_ (with BAr_F _= tetrakis[3,5‐bis(trifluoromethyl)phenyl]borate), with a Rh:monomer ratio of 1:2, **BTA *m*‐P(*S*),(*S*)** promoted the hydrogenation of dimethyl itaconate at room temperature, furnishing the corresponding hydrogenation product in 82% ee (Figure 12c). The enantiomeric ligand, **BTA *m*‐P(*R*),(*R*)**, resulted in similar selectivity favoring the product with opposite configuration. Remarkably, the active rhodium catalytic centers and stereogenic centers are separated by 12 covalent bonds. The control experiment employing ^Et^ **BTA *m*‐P(*S*),(*S*)**, the analogue of **BTA *m*‐P(*S*),(*S*)** with two ethylated amide functions, gave no selectivity for the reaction as a consequence of its inability to form the supramolecular helices. Likewise, no selectivity was observed for **BTA *m*‐P(*S*),(*S*)** in DCM, a solvent in which BTA monomers are molecularly dissolved. These experiments demonstrate that supramolecular helical BTA assemblies are enantioselective, not the monomers. This study paves the way for the use of supramolecular chirality of SP as the sole contributor for dictating the selectivity in organometallic processes. Furthermore, a catalytically inactive enantiopure BTA comonomer, **BTA (*S*)**, possessing two n‐octyl and one chiral 1‐methylheptyl side chains, was engaged as a comonomer for the same reaction, resulting in an increased selectivity (88% ee). This enhanced catalytic performance confirms that modulation of the chiral BTA helical catalysts by combining several types of BTA monomers is feasible, thus offering the possibility to optimize catalytic processes at low synthetic cost.

## 3 Supramolecular Polymer Catalysts Made From Achiral Catalytically–active Monomers

The 1D coassembly of several monomers or components into a chiral SP makes it possible to use achiral monomers embedding catalytic sites in asymmetric catalysis. The main challenges are (i) to avoid the presence of dissociated achiral monomers that would inevitably lower the selectivity of the reaction by means of a nonstereoselective catalytic route, (ii) to get an efficient chirality transfer/induction process from the supramolecular chiral helices to the intrinsically achiral catalytic sites, and (iii) to provide a sufficiently strong stereodiscriminating environment to the catalytic site in order for the reaction to proceed with significant stereoselectivity. While it may appear at first sight irrelevantly challenging, the approach also presents several advantages: (i) the impact of supramolecular chirality on the observed selectivity can be firmly assessed since the individual (dissociated) components are not enantioselective, (ii) various chemical or physical sources of chirality can be used, (iii) a small amount of chiral inducers can be sufficient to provide a stereodiscriminating environment to several catalytic sites thanks to the “S&S” effect, as explained in the introduction of this review, (iv) the catalytically‐active component and the chiral component can be designed independently, and the final system can be optimized in a combinatorial approach. In the next paragraphs, we will present examples related to this topic that rely either on SMSB (part 3.1) or on chiral monomers (part 3.2) to induce a chiral environment to achiral catalytically active monomers.

### 3.1 Through SMSB

SMSB arises inherently from the internal properties and dynamic interactions of the system, leading to the destabilization of the symmetric racemic state under nonequilibrium steady states.^[^ ^136^ ^]^ As a result, the system preferentially leads to the generation of one enantiomer over the other. If a statistically relevant number of experiments is conducted, and in the strict absence of chiral bias, the result of the SMSB process is nevertheless stochastic; that is, the two opposite chiral states are obtained the same number of times. In molecular systems, the occurrence of SMSB is rare, and the Soai reaction^[^ ^89^ ^]^ is probably the only existing example reported to date. SMSB is more frequent for supramolecular assemblies in crystalline and solid states as well as in SPs for which chiral structures emerge spontaneously within assemblies of otherwise achiral monomeric units. Subtle environmental factors, such as temperature, solvent polarity, or shear forces, can trigger a supramolecular polymer system to result in a chiral form. However, utilizing SMSB‐type SPs to catalyze asymmetric reactions is still a big challenge, with only two examples of this new class of absolute asymmetric catalysts^[^ ^136^ ^]^ reported to date.

**Figure 13:**
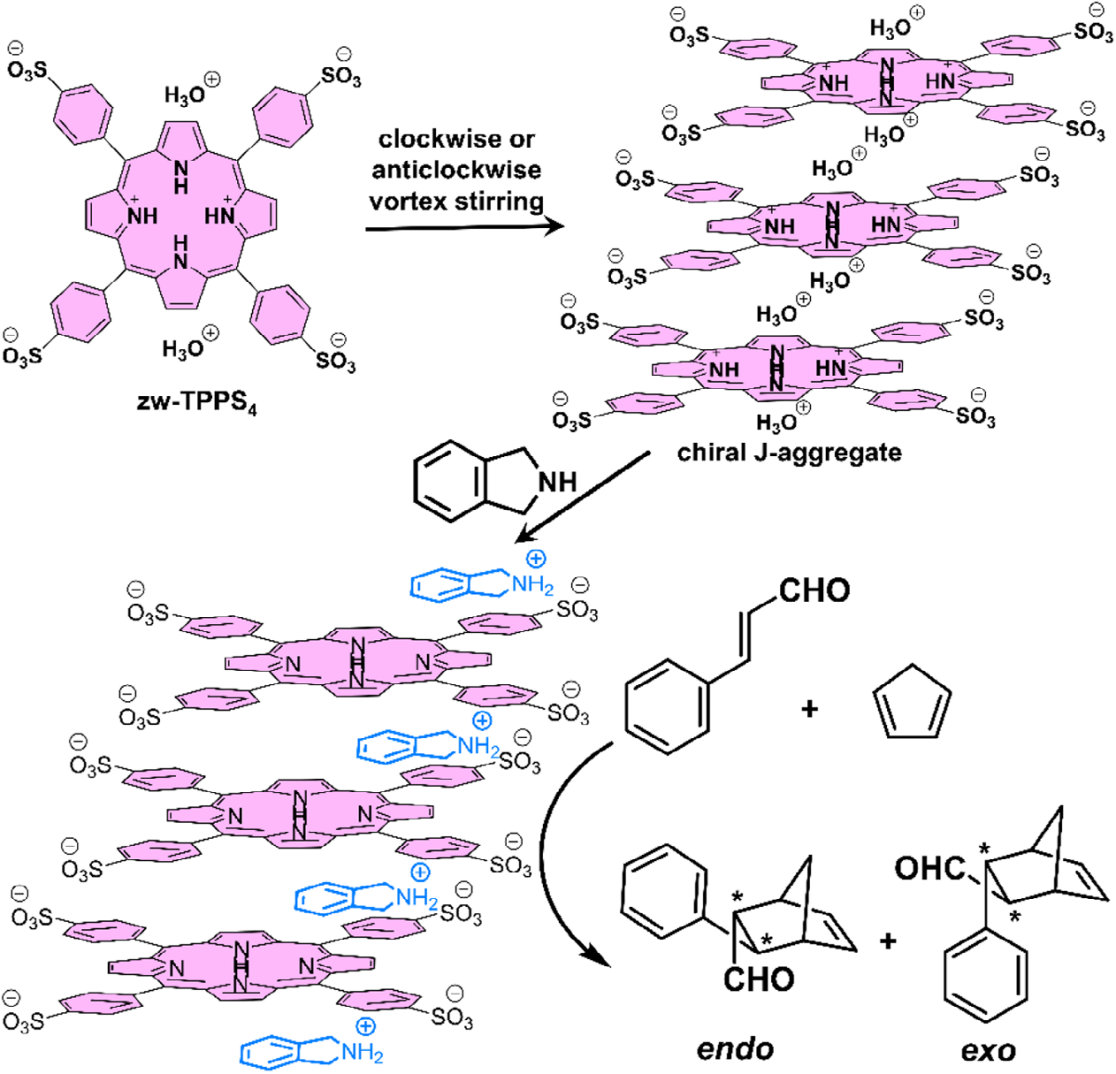
Chemical structure of **zw‐TPPS_4_** (zw stands for zwitterionic), its assembly to chiral J‐aggregate upon vortex stirring, and the implementation of the hybrid assemblies formed in the presence of isoindoline as a catalyst for a Diels‐Alder reaction.

As a proof of the concept, Moyano and coworkers^[^ ^137^ ^]^ selected the achiral amphiphilic porphyrin compound, *meso*‐tetrakis(4‐sulfonatophenyl)porphyrin (**zw‐TPPS_4_**, Figure 13), known for its capability to form well‐defined J‐aggregates with biased chirality through SMSB induced by hydrodynamic forces in water (clockwise or counterclockwise vortex stirring).^[^ ^138^ ^]^ The Diels–Alder cycloaddition between (*E*)‐cinnamaldehyde and cyclopentadiene in water, catalyzed by amines, was chosen because cationic intermediates were anticipated to interact with the surface of the J aggregates occupied by anionic sulfonate groups. Both pristine **zw‐TPPS_4_** and hetero‐aggregates between **zw‐TPPS_4_** and various achiral secondary amines, yielded the Diels‐Alder product in a nonracemic form, albeit with very low ee values (5.9 ± 0.6% ee at best). Reactions proceeded in higher yields in the presence of the secondary amines thanks to the formation of a well‐defined iminium ion between the amine and (*E*)‐cinnamaldehyde. In order to formally assess the origin of the observed selectivity, the handedness of the hetero‐aggregates between **zw‐TPPS_4_** and isoindoline was selected thanks to the addition of a chiral selector (a chiral quaternary ammonium cation), used in small amount relative to **zw‐TPPS_4_**, and probed by CD. The observed selectivity correlates with the handedness of the J aggregates, that is, (2*S*,3*S*)‐*exo*/(2*R*,3*R*)‐*endo* and (2*R*,3*R*)‐*exo*/(2*S*,3*S*)‐*endo* pairs formed preferentially when the J aggregates exhibit bisignated (‐/+) and (±) CD spectra, respectively. A control experiment rules out a direct influence of the chiral selector on the selectivity, confirming the crucial role played by the helical J aggregates in generating the small chiral imbalances detected in the *endo* and *exo* isomers of the Diels‐Alder product. This constitutes a unique example for which a truly chiral macroscopic force (hydrodynamic vortices) can be converted into biased chirality at the molecular scale, thus affording top‐bottom chirality transfer.^[^ ^73^ ^]^

**Figure 14:**
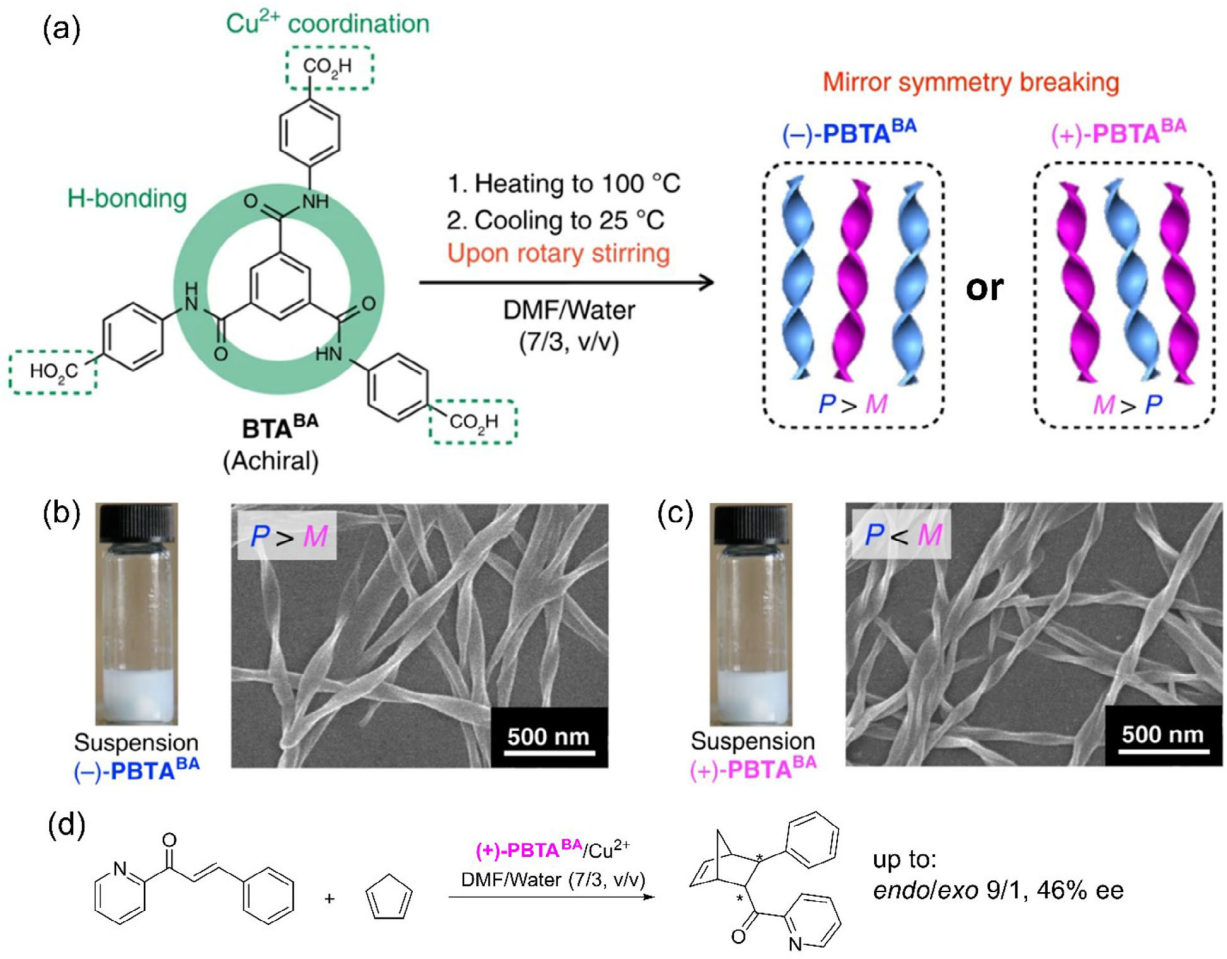
a) Chemical structure of **BTA^BA^** and its assembly into biased helical assemblies **(–)‐PBTA^BA^** and **(+)‐PBTA^BA^** thanks to SMSB. b),c) Photographs of the suspensions obtained upon cooling and rotary stirring of the heated solutions of **BTA^BA^**. SEM images of the air‐dried suspensions corresponding to *P*‐and *M*‐biased supramolecular helical assemblies. d) Catalytic performance in the copper‐catalyzed Diels‐Alder reaction. Figures a)‐c) are adapted from Ref. [139] https://doi.org/10.1038/s41467‐019‐11840‐3, under the terms of the Creative Commons CC BY license https://creativecommons.org/licenses/.

The innovative approach of combining SMSB‐induced formation of SP with preferred handedness and asymmetric catalysis was also implemented by Liu and coworkers in 2019.^[^ ^139^ ^]^ Building on previous observations,^[^ ^140^ ^]^ the authors found that the structurally–simple BTA monomer, **BTA^BA^** (Figure 14a), designed with peripheral benzoic acid groups for metal coordination, yields helical nanoribbons with a preferred handedness under specific conditions. When a solution of **BTA^BA^** in dimethylformamide (DMF) and water was heated and then cooled down upon rotary stirring, vortex stirring, or sonication, it assembled through SMSB, producing either preferentially right‐handed (*P*) or left‐handed (*M*) helical nanoribbons (Figure 14b–c). Rotary stirring played a critical role in this process, with the handedness of the nanoribbons being determined stochastically but independently from the stirring direction. These nanoribbons were mixed with Cu(NO_3_)_2_ and the resulting metal hybrid SPs were evaluated in the Diels–Alder reaction between azachalcone and cyclopentadiene (Figure 14d). The major *endo* product for 76 independent runs was always obtained with significant ee (> 11%), the configuration of which correlates well with the preferred handedness of the nanoribbons. Overall, a satisfying stochastic distribution of the ee values is observed with average ee values of 18.3 ± 3.9% and 17.5 ± 2.8% for the (*S*) and (*R*) enantiomers, respectively, of the endo product. However, the variations in the ee cannot be correlated with the CD intensity. Optimizing the Cu^2+^:**BTA^BA^** ratio to 1/10 and adding NaOH led to an improved enantioselectivity (46% ee), a remarkable value for a system devoid of any chiral inducers. Induced CD signal could not be detected for copper but was found for methylene blue, which was electrostatically bound to the carboxylate groups of the nanoribbons. This work unambiguously reveals the possibility to implement helical nanoribbons formed through SMSB as a suitable chiral environment for asymmetric catalysis.

### 3.2 Through the “S&S” Effect

Controlling the main chain chirality of covalent or SPs by mixing a few enantiopure monomers (called “the sergeants”) and a large number of achiral ones (called “the soldiers”) is far more widespread in the literature than through the aforementioned SMSB process.^[^ ^39^, ^40^, ^42^ ^]^ As explained in the introduction, this strategy takes advantage of the ability of the “sergeants” to induce their chiral preference in several “soldiers” through the propagation of a conformational bias through the whole SP. The extent of the “S&S” can be quantified by means of numerical^[^ ^141^ ^]^ and statistical models.^[^ ^142^ ^]^ The latter model allows extracting the energetic parameters associated with the chiral defects present in the supramolecular assemblies: the helix reversal penalty (HRP), which corresponds to the energy needed to reverse the handedness of the supramolecular helix, and the mismatch penalty (MMP), which is the energy paid for incorporating a “sergeant” in a helix of its nonpreferred helicity.^[^ ^143^ ^]^ It is currently accepted that a high “S&S” effect can be reached for a high HRP value and a low (but not null) MMP value. Helically biased SPs operating through the S&S effect have been applied in chirality‐driven applications such as CPL emitters,^[^ ^144^ ^]^ and spin‐selective systems,^[^ ^145^ ^]^ but prior to our work, no example had been provided in the field of asymmetric catalysis.

**Figure 15:**
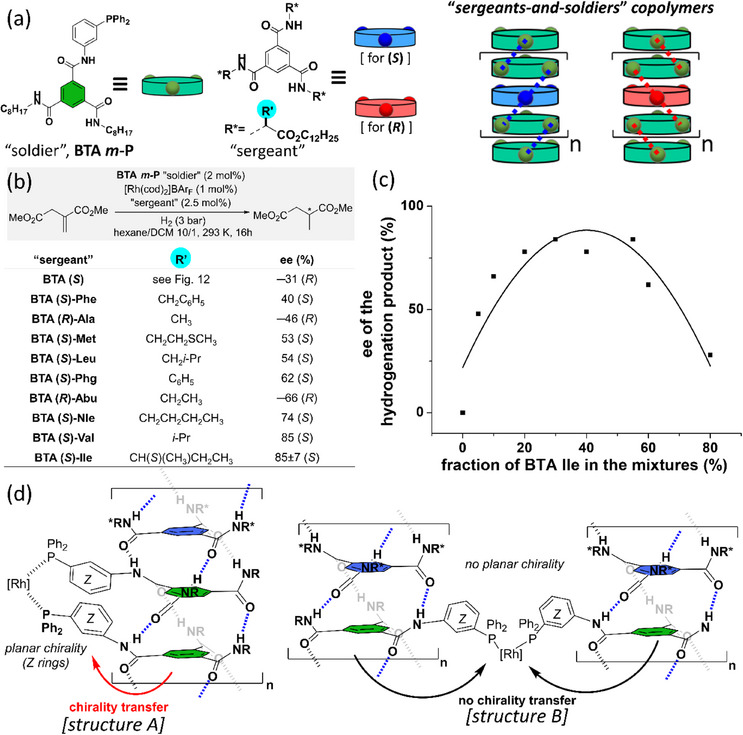
a) Chemical structure of the BTA monomers and schematic representation of their coassembly into “S&S” type SCPs. “Sergeants” in blue and red induce the preferential formation of right‐handed and left‐handed helices, respectively (represented by the dotted lines connecting the rings). The dots on the rings represent the amide functions (three per monomer). b) Catalytic performance of the S&S‐type copolymers in the rhodium‐catalyzed hydrogenation reaction. c) Plot of the enantioselectivity as a function of the fraction of the “sergeant” (**BTA Ile**) present in the mixture. The fraction of “sergeants” is equal to the concentration in “sergeants” divided by the total concentration in BTA monomers; in that case, [**BTA Ile**]/([**BTA *m*‐P**]+[**BTA Ile**]). This definition is valid throughout this review and abbreviated as fs. d) Schematic representation of the postulated structures leading to highly (structure A) and poorly (structure B) selective catalytic systems.

In a preliminary attempt,^[^ ^135^ ^]^ the achiral BTA phosphine ligand, **BTA *m*‐P**, was mixed with the chiral BTA comonomer, **BTA (*S*)**, in a 1/1.25 ratio and evaluated in the aforementioned model rhodium‐catalyzed hydrogenation reaction (Figure 15a,b). A modest but significant selectivity of 31% ee was obtained for the hydrogenation product, which stems solely from the coassembly of the two monomers in the same copolymer. This motivated us to investigate other “sergeants,” since we anticipated that the structure of the “sergeants” plays a key role in the coassembly and chirality induction processes.

We thus prepared a library of chiral ester BTA comonomers on account of their convenient accessibility and straightforward synthesis from amino acids.^[^ ^50^, ^146^ ^]^ Catalytic mixtures between **BTA *m*‐P**, the “soldier,” coordinated to [Rh(cod)_2_]BAr_F_ (Rh to **BTA *m*‐P** ratio of 1/2) and various ester BTAs, the “sergeants,” were evaluated in the hydrogenation reaction.^[^ ^147^ ^]^ In the initial screening, the ratio between the “sergeant” and the “soldier” was again fixed to 1/1.25. All ester BTAs provided significant selectivity, with **BTA Val** and **BTA Ile** yielding the highest ee values (85%, Figure 15b). The control experiment again demonstrated that the enantioselectivity of the catalytic reaction stemmed from supramolecular helical coassemblies. Importantly, the observed enantioselectivity with the couple **BTA *m*‐P**/**BTA Ile** proved to be well reproducible (85 ± 7% ee). The influence of the molecular structure of the “sergeant” on the enantioselectivity will be discussed later in this part.

Furthermore, the influence of the amount of **BTA Ile**, an efficient enantiopure BTA comonomer, on the selectivity of the catalytic reaction was probed. It was found that the fraction of **BTA Ile** relative to the “soldier” can be reduced to 20%, which corresponds to 1 “sergeant” for 4 BTA ligands or 2 Rh centers, without compromising the enantioselectivity (Figure 15c). Crucially, the nonlinear increase of the enantioselectivity as a function of the fraction of “sergeants” unambiguously unveiled that chirality induction occurred through the S&S effect in these supramolecular helical coassemblies. The S&S effect was also demonstrated for helical covalent polymers implemented in asymmetric catalysis.^[^ ^83^, ^148^, ^149^, ^150^ ^]^ However, this BTA system constituted at that time the first example for which a sub‐catalytic amount of a chiral inducer can be used in asymmetric catalysis without decreasing the selectivity. Surprisingly, the enantioselectivity eroded for the mixtures containing a fraction of “sergeants” superior to 55% (Figure 15c). The structure of the helical coassemblies coordinated to Rh, which proved to be insoluble under our reaction conditions, was thoroughly characterized by means of FT‐IR and CD analyses. The decreased selectivity was not caused by the diminished optical purity of the co‐assemblies but rather by a change in the coordination mode of the metal center. A rational mechanism of the chirality induction was thus proposed that goes from the stereogenic centers in the side chains of the “sergeant,” to the biased supramolecular helicity of the polymeric scaffold and from there to the catalytic rhodium centers. Presumably, the presence of a large fraction of “sergeants” prevents the coordination of the Rh center by two neighboring ligands belonging to the same helix, which leads to a crosslinked structure for which efficient chirality transfer to the unit close to the Rh center is hampered (Figure 15d, structure B). Alternatively, the chelation of the rhodium centers by two **BTA *m*‐P** ligands belonging to the same helix is expected to be favored when fewer “sergeants” are present (Figure 15d structure A). Only in that case, efficient catalytic induction can arise from the helically biased polymer backbone to the connecting rings (labeled as rings *Z* in structures [A] and [B]) facing one another with their *Si,Si* (or *Re,Re*) faces, and from there to the peripheral PPh_2_ units, which may chelate the rhodium atom in the favorable *C*_2_ quadrant configuration.

**Figure 16:**
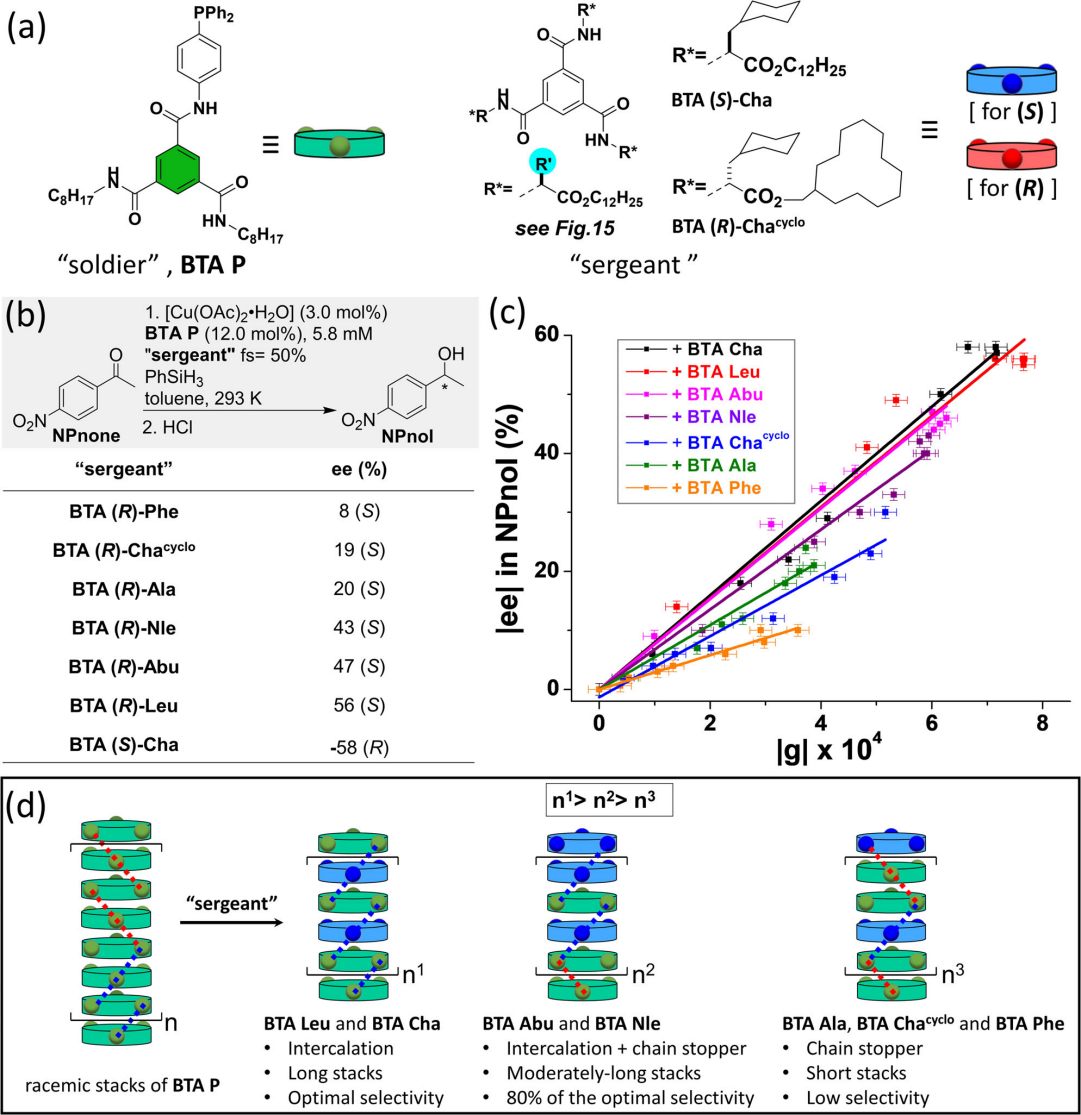
a) Chemical structure of the BTA monomers (see also Figure 15a). b) Catalytic performance in the copper‐catalyzed hydrosilylation reaction. c) Plot of the enantiomeric excess in **NPnol** versus the Kuhn anisotropy factor extracted from CD analyses of the “S&S” type coassemblies. d) Schematic representation of the role of the “sergeant” as intercalator or chain stopper depending on its structure. Chiral defects are represented by the reversal of the handedness of the helical coassemblies.

While this initial study unambiguously exemplifies the potential of implementing the S&S effect in asymmetric catalysis, we turned out to probe a new catalytic reaction for which the supramolecular helical catalyst would be more soluble and thus easier to tune. Mixtures between achiral BTA ligands (the “soldiers”) and various ester BTAs (the “sergeants”) were thus evaluated in a 1:1 ratio in the copper‐catalyzed hydrosilylation of 4‐nitroacetophenone (**NPnone**).^[^ ^151^ ^]^ This reaction, conducted with phenylsilane as the reducing agent, became our model reaction to probe the S&S effect in catalysis. Here, the BTA ligand with a *p*‐phenylene linker, named **BTA P** (Figure 16a) in the following, was found to be more efficient than **BTA *m*‐P**. “Sergeants” with a branched alkyl chain connected to the stereogenic center, **BTA Leu** and **BTA Cha**, provided the best selectivities under the conditions shown in Figure 16b (56–58% ee). Control experiments similar to the ones conducted above for the Rh catalyst confirm that the selectivity comes from the supramolecular chirality of the coassemblies between the BTA ligand and the “sergeant.” However, in this case, the coassemblies remain soluble during the whole catalytic process, probably because the expected active species located at the periphery of the supramolecular helices, a phosphine copper hydride complex,^[^ ^152^ ^]^ does not compromise the solubility of the supramolecular system, a key point for a precise characterization of the system and the development of dynamic switchable catalysts (see part 4).

The nature of the “sergeant” has actually a great influence on the enantioselectivity, which varies from 8% ee for the worst “sergeant” to 58% ee for the best ones (Figure 16b). Seven “sergeants” were selected spanning the whole observed selectivity range, **BTA (*R*)‐Leu**, **BTA (*S*)‐Cha**, **BTA (*R*)‐Nle**, **BTA (*R*)‐Abu**, **BTA (*R*)‐Ala**, **BTA (*R*)‐Cha^cyclo^,** and **BTA (*R*)‐Phe**, in order to better apprehend the relation between the structure of the supramolecular copolymer and the enantioselectivity.^[^ ^153^ ^]^ Precatalytic mixtures, obtained by combining **BTA P**, [Cu(OAc)_2_], and various quantities of one of the “sergeant” were characterized by FT‐IR, CD, and SANS analyses. Firstly, the extent of the S&S effect was found to be quite low for all these “sergeants,” that is, the optimal enantioselectivity was reached only for a fraction of “sergeant” ≥ 40%; this point will be commented further below. Secondly, the enantioselectivity of the reaction correlates satisfactorily with the Kuhn anisotropy factor extracted from the CD measurements, which reflects the net helicity of the coassemblies (Figure 16c). This means that there is a direct link between the selectivity and the optical purity of the supramolecular helical coassemblies. Thirdly, the optical purity increases in all cases as a function of the fraction of “sergeants” but reaches a different plateau value depending on whether the coassemblies are single‐handed, exhibit very low optical purity, or show an intermediate behavior. After determining the composition and length of the SCPs, the role of the “sergeant” can be dissected for these three classes as followed: (i) “sergeants” that intercalate strongly with **BTA P** and lead to the optimal selectivity (case of **BTA Leu** et **BTA Cha**), (ii) those that intercalate but also play the role of chain capper and give intermediate selectivity (**BTA Abu** et **BTA Nle**), and (iii) those that mainly act as chain capper and display low selectivity (Figure 16d). Controlling the composition and structure of copolymers assembled through a cooperative pathway as for BTA monomers is difficult because of their dynamic nature and the difficulty of tuning the interaction energies between the monomers.^[^ ^154^ ^]^ This constitutes the first example for which the structure of S&S‐type copolymers is tuned by the nature of the “sergeant” with direct implication on their catalytic performance.

**Figure 17:**
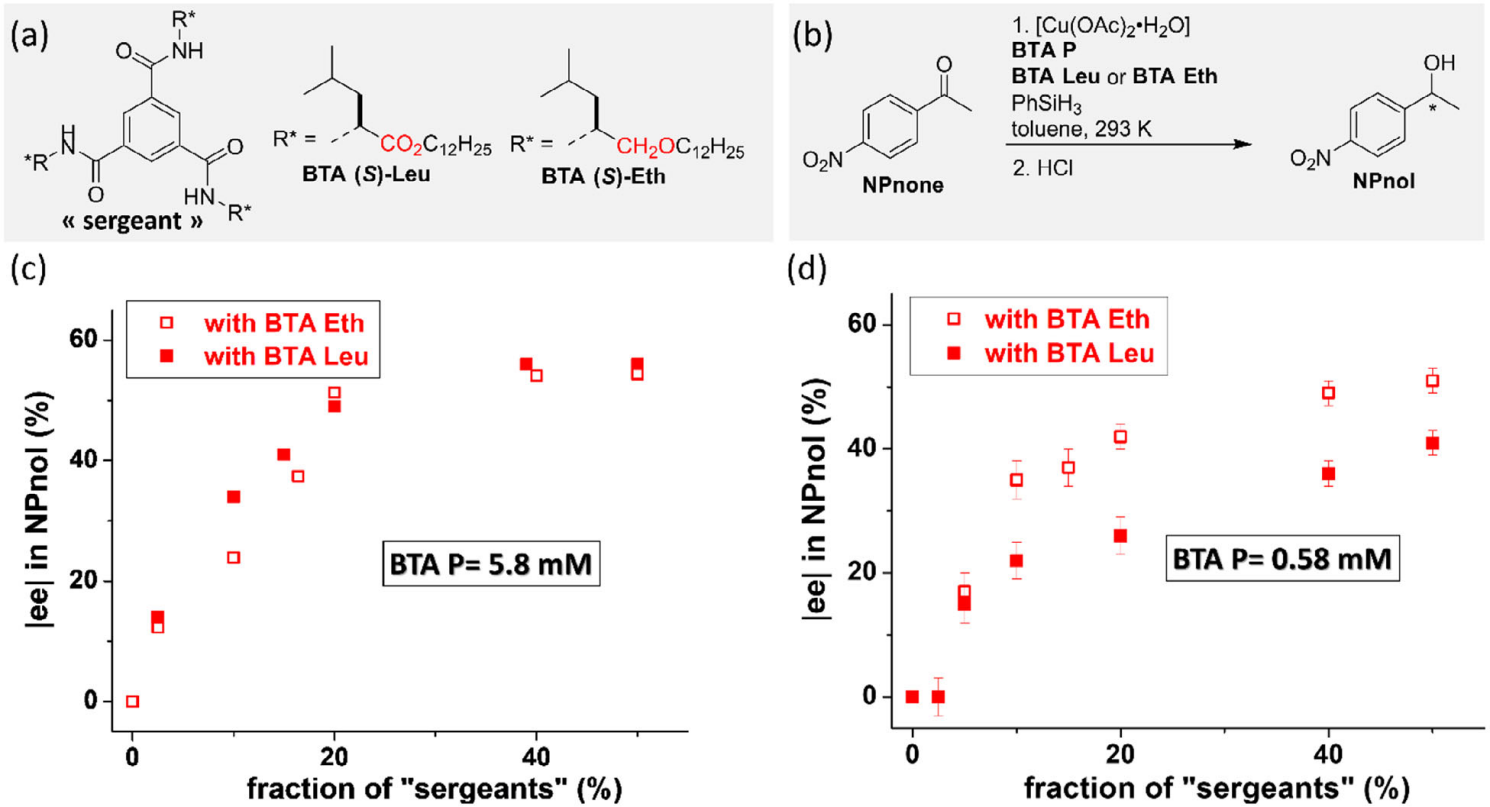
a) Chemical structure of the BTA monomers **BTA (*S*)‐Leu** and **BTA (*S*)‐Eth**. b)‐d) Catalytic performance in the copper‐catalyzed hydrosilylation reaction for the “S&S” type coassemblies for two different concentrations in **BTA P**.

Tuning the structure of the “sergeant” is thus of paramount importance to improve the performance of supramolecular helical catalysts. However, even though **BTA Leu** and **BTA Cha** efficiently coassemble with and furnish a stereodiscriminating environment to **BTA P**, the optimal selectivity is reached only for a high fraction of “sergeant”. Likewise, it was established in our previous studies^[^ ^50^, ^146^, ^155^ ^]^ that **BTA Leu** and **BTA Cha** preferentially form dimeric structures on their own, with BTA arms connected by hydrogen bonds involving the ester carbonyl functions, not the amide ones. It was anticipated that these structures can sequester part of the **BTA P** monomers and thus decrease the efficiency of chirality induction to potent catalytic helical BTA copolymers. Replacing the ester group of **BTA Leu** by an ether function, yields **BTA Eth** (Figure 17a); a monomer that forms strong homoassemblies by itself and intercalates with other “soldiers” very efficiently. Analytical data reveal that coassemblies between **BTA P** and **BTA Eth** are indeed more stable than those with **BTA Est** because the generation of competitive species is minimized (or destabilized). When the respective mixtures were applied to catalysis, we found that **BTA Leu** and **BTA Eth** behave similarly when the concentration of **BTA P** is high, but a significantly improved selectivity was observed for **BTA Eth** mixtures at a lower concentration (Figure 17b–d).^[^ ^156^ ^]^ Even though this “sergeant” design does not improve the extent of the “S&S” effect, it allows to perform our model hydrosilylation at a lower concentration in helical coassemblies, thus decreasing the catalytic loading without jeopardizing the enantioselectivity. This observation agrees with our previous work showing that optimal selectivity is reached only when sufficiently long coassemblies are present.^[^ ^157^ ^]^

**Figure 18:**
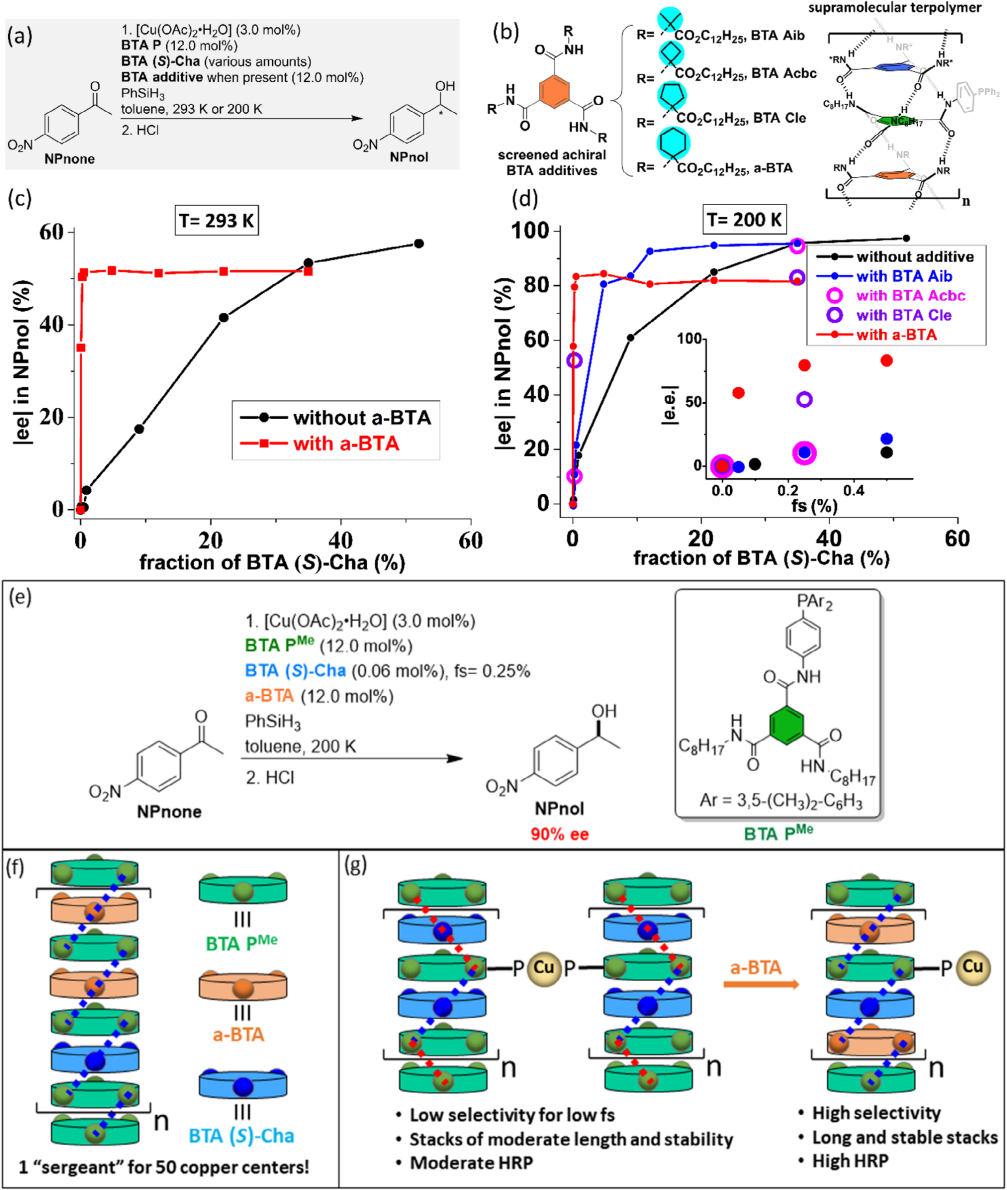
a) Reaction scheme and conditions for probing the influence of achiral additives on the extent of the S&S effect displayed by BTA helical catalysts. b) Molecular structures of the tested achiral BTA additives and schematic representation of the BTA helical catalyst embedding the three types of monomers (BTA ligand, “sergeant,” and additive). c) Plot of the ee in **NPnol** as a function of the fraction of “sergeants” for the S&S‐type helical catalyst composed of **BTA P** and **BTA Cha** in the presence and absence of **a‐BTA**. d) Plot of the ee in **NPnol** as a function of the fraction of “sergeants” for the S&S‐type catalyst composed of **BTA P** and **BTA Cha** in the presence and absence of different achiral BTA additives. e) Best‐performing S&S‐type helical catalyst in the copper‐catalyzed hydrosilylation reaction. f) Representation and composition of the best‐performing supramolecular helical catalyst for the hydrosilylation reaction. g) Schematic representation of the role of the achiral BTA additive, **a‐BTA**.

Various parameters were screened with the aim of drastically improving the extent of the “S&S” effect, with **BTA P** and **BTA Cha** as the selected “soldier” and “sergeant,” respectively. The impact of the presence of the copper coordinated at the periphery of the supramolecular helices was probed, and we demonstrated that copper tends to crosslink the helices. However, these crosslinks are reversible and do not form when the fraction of “sergeant” is superior to 20% in the stacks (for a representation of these crosslinks, see Figure 18g).^[^ ^158^ ^]^ Anyway, varying the amount of copper does not significantly improve the extent of the S&S effect, which remains very modest under these conditions. Assuming that this low S&S effect might be due to the *C*_2_‐symmetry of **BTA P**, we envisaged that achiral additives may remove chiral defects in the coassemblies. Inspired by the ability of α quaternary amino acid to stabilize the alpha helix conformation of peptides, we probed the influence of various achiral BTA monomers derived from α,α’‐disubstituted amino esters (Figure 18ab). At 293 K, the addition of **a‐BTA**, the BTA monomer derived from 1‐aminocyclohexane carboxylic acid, led to an enhancement of the S&S effect by two orders of magnitude: 0.5% (relative to **BTA P**) or 0.25% (relative to all BTA monomers present in the mixture) of “sergeant” is now enough to get a selectivity of 51% ee, close to the optimal selectivity of the system (58% ee, Figure 18c).^[^ ^159^ ^]^ Several achiral additives were compared at 200 K, a temperature for which the optimal selectivity is increased to 97% ee for the supramolecular helical catalyst without additive. All tested achiral BTA monomers derived from α,α’‐disubstituted amino esters led to an increase in the selectivity at low “sergeant” ratio (Figure 18c). Despite their very similar structures, these additives afforded different levels of S&S effect improvement and maximal selectivities, with **a‐BTA** showing the highest enhancement of the S&S effect but at the cost of a slightly decreased selectivity (ee max of 82%). Our in‐depth study reveals that playing on the nature of the additive and its amount offers a good compromise to tune the extent of the S&S effect without jeopardizing the optimal selectivity of the terpolymer in our catalytic reaction of reference.^[^ ^160^ ^]^ Furthermore, the remarkable effect of **a‐BTA** is also observed with **BTA P^Me^**, an analogue of **BTA P** with aryl rings substituted by methyl groups at the 3 and 5 positions (Figure 18e). With this ligand, the product was obtained in 90% ee (200 K) with as low as 0.25% of “sergeants;” conditions under which the coassemblies without additive exhibited no significant selectivity (< 3% ee). This means that one molecule of “sergeant” is able to furnish a stereodiscriminating environment to 50 copper atoms; this is the best‐performing S&S catalyst, surpassing all existing supramolecular and covalent helical catalysts^[^ ^83^, ^148^ ^]^ reported to date (Figure 18f).

Analytical data were collected to help rationalize the counterintuitive influence of **a‐BTA**. It was found that the role of **a‐BTA** is mainly to decrease the number of chiral defects present in the coassemblies; that is, it exerts a long‐range conformational control. The HRP doubled from 11.0 to 22.1 kJ mol⁻¹, indicating that the helices become much more resistant to reversals. These findings align well with the observed increased thermal stability and rigidity of the helical terpolymers incorporating **a‐BTA**. The slight variation in the optimal selectivity might be explained by a conformational change occurring close to the catalytic site.

**Figure 19:**
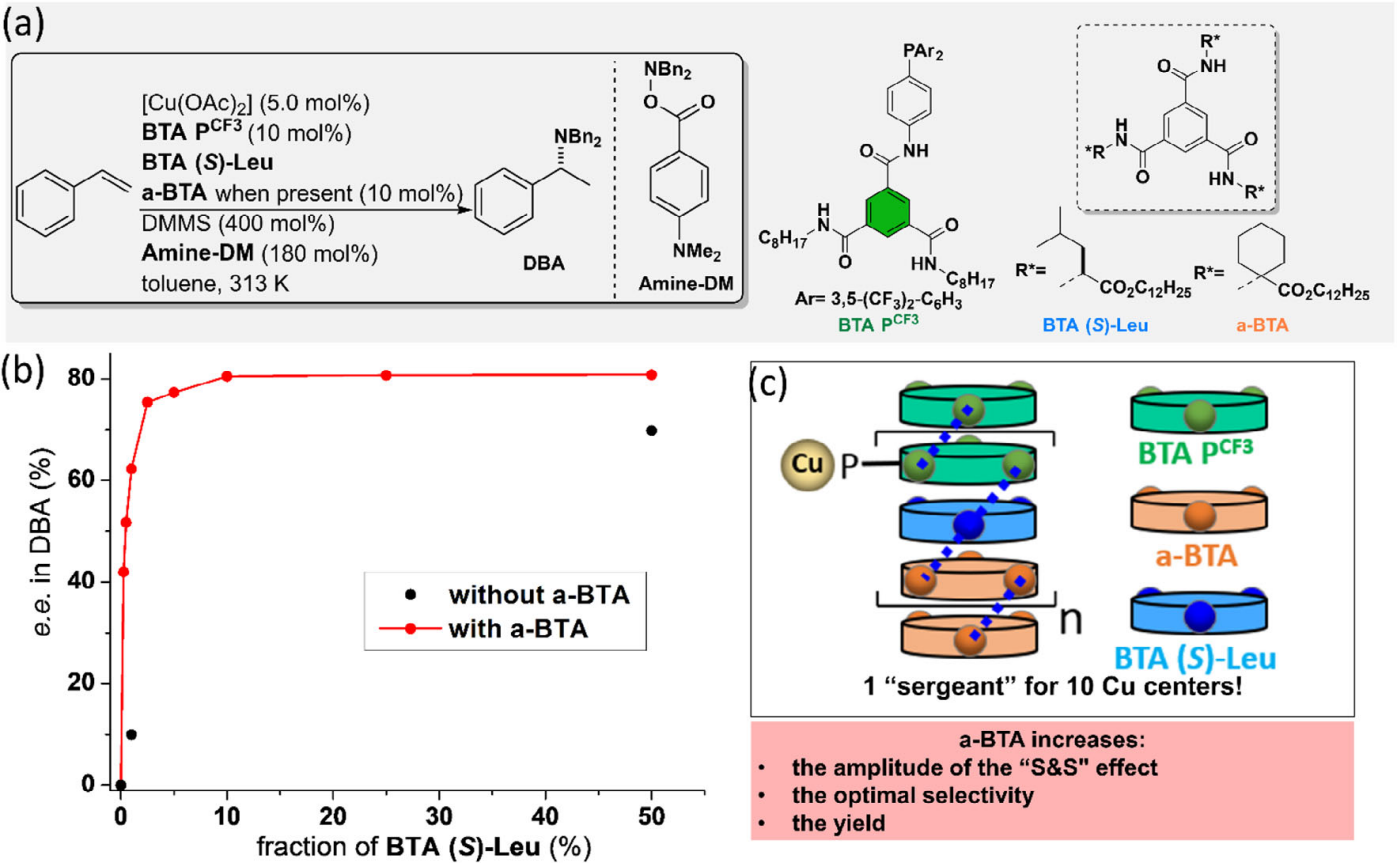
a) Reaction scheme and conditions for the copper‐catalyzed hydroamination with BTA helical catalysts. b) Plot of the ee in **DBA** as a function of the fraction of “sergeants” for the S&S‐type helical catalyst composed of **BTA P^CF3^** and **BTA (*S*)‐Leu** in the presence and absence of **a‐BTA**. c) Schematic representation of the structure of the helical BTA terpolymer used for the hydroamination reaction as well as the benefits brought by the presence of **a‐BTA**.

The scope of this highly efficient S&S‐type supramolecular catalyst was further probed in the Cu‐H catalyzed hydroamination of styrene, a reaction that requires an amine electrophile (**Amine‐DM**, Figure 19a).^[^ ^161^ ^]^ Various BTA ligands were screened as “soldiers”; the one with aryl groups substituted by CF_3_ moieties at the 3 and 5 positions, **BTA P^CF3^**, emerged as the optimal ligand. Under optimized conditions (Figure 19a), the coassemblies consisting of an equimolar mixture of **BTA P^CF3^** and **BTA Leu** furnished the hydroamination product in 93% NMR yield and 69% ee. The achiral BTA additive, **a‐BTA**, again had a drastic influence on the catalytic performance. The product was now obtained in 75% ee and 95% NMR yield with as low as 2.5% of sergeant, conditions under which the pristine coassembly display far lower ee and yield (Figure 19b). One “sergeant” controls 10 copper centers, which is still impressive but lower than for the aforementioned hydrosilylation reaction,^[^ ^159^ ^]^ probably because of the higher temperature of the catalytic reaction (313 K). Remarkably, the achiral additive not only improves the extent of the S&S effect but also significantly improves the yield and optimal enantioselectivity, further demonstrating the key advantages of using achiral additives to regulate the performance of BTA‐based supramolecular helical catalysts.

## 4 Stereochemically Switchable Supramolecular Helical Catalysts

One particularly interesting property of SPs is their inherent dynamicity and reversibility, which makes it possible to easily tune their structures and properties by means of suitable stimuli. This is particularly prominent for hydrogen‐bonded SPs^[^ ^162^ ^]^ since their composition and length can be modulated by the addition of complementary monomers or chemical triggers. The dynamic nature of BTA‐based supramolecular helical catalysts makes them attractive candidates for controlling the outcome of catalytic reactions in response to external stimuli. Our efforts to elaborate stereochemically switchable catalysts based on this concept are summarized in this part.

### 4.1 For Modulating the Enantioselectivity

A multi‐configurable supramolecular catalyst that can be predictably switched between different functional states presents a significant advantage over conventional molecular catalysts. To investigate the reversible properties of BTA‐based supramolecular systems, we first explored the possibility of modulating the catalyst enantioselectivity by varying the length of the helices. This was achieved by adding anions, since they are known to act as chain stoppers of hydrogen‐bonded SPs, thus enabling to reduce their average length without affecting their structure.^[^ ^163^ ^]^

Our findings revealed that the addition of chloride anion, under the form of its tetraphenylphosphonium (TPP) salt, a noncompeting cation, gradually reduced the enantioselectivity of the model hydrosilylation reaction, promoted by the S&S mixture between **BTA P** and **BTA Cha**.^[^ ^157^ ^]^ The selectivity even dropped to 0% ee when one equivalent of TPPCl relative to **BTA P** was used. This suggested that the helices had been shortened to such an extent that the copper centers were no longer positioned in a suitable chiral environment. Notably, TPPCl did not act as a simple on‐off switch for selectivity but allowed for programmable modulation of enantioinduction, with selectivity decreasing stepwise as more chloride ions were added to the catalytic mixture.

**Figure 20:**
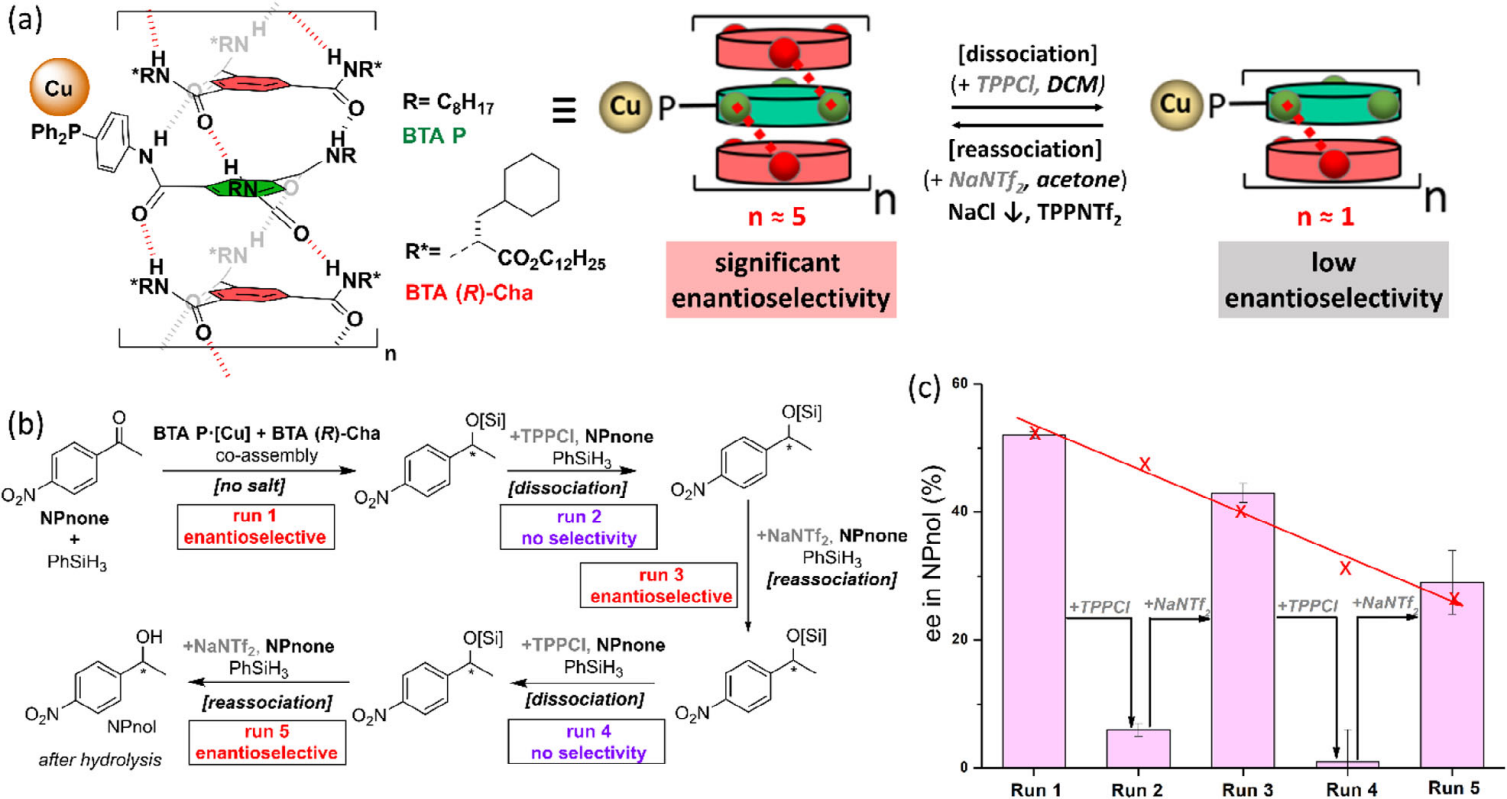
a) Schematic representation of the strategy used to reversibly dissociate supramolecular helical catalysts together with the relationship between their length and their selectivity toward the model hydrosilylation reaction. b) Reaction scheme for the successive transformation of several equivalents of **NPnone** and the selectivity expected for each run. c) Plot of the obtained enantioselectivity in **NPnol** for the different runs.

Furthermore, we envisioned removing the chloride anion in the solution phase via a salt metathesis reaction. Reassembly of the helices was achieved through the addition of sodium triflimide (NaNTf_2_), which facilitated the removal of the chloride ions by co‐precipitation. It was anticipated that this process would restore the length of the helices and, consequently, the enantioselectivity of the catalytic system (see the schematic representation of the concept in Figure 20a). Five successive runs of the model hydrosilylation reaction were conducted, which consisted to two disassembly/reassembly cycles achieved by adding TPPCl (in dichloromethane) in runs 2 and 4 and NaNTf_2_ (in acetone) in runs 3 and 5 (Figure 20b). The significant decrease in enantioselectivity observed in run 2 (6 ± 1% ee) and run 4 (0 ± 5% ee) indicated that helix disassembly occurred in the presence of TPPCl, while the restored selectivity in run 3 (43 ± 2% ee) and run 5 (29 ± 5% ee) upon the addition of NaNTf_2_ demonstrated the successful implementation of our concept (Figure 20c). The reassembled helices exhibited significant enantioselectivity, yet lower than that of the initial state (before disassembly). Spectroscopic analyses supported this observation, showing that the reassembled helices were shorter than the pristine ones, likely due to the polar nature of the solvents used to dissolve the salts. Determination of the average length of the coassemblies indicated that a critical length in the range of ten monomer units was actually required to locate most of the copper centers in a chiral environment suitable for asymmetric induction. As a perspective of this work, it would be particularly attractive to commute the selectivity of this catalyst during a polymerization process in order to form structurally complex multi‐stereoblock copolymers.^[^ ^164^ ^]^

### 4.2 For Commuting the Enantioselectivity

**Figure 21:**
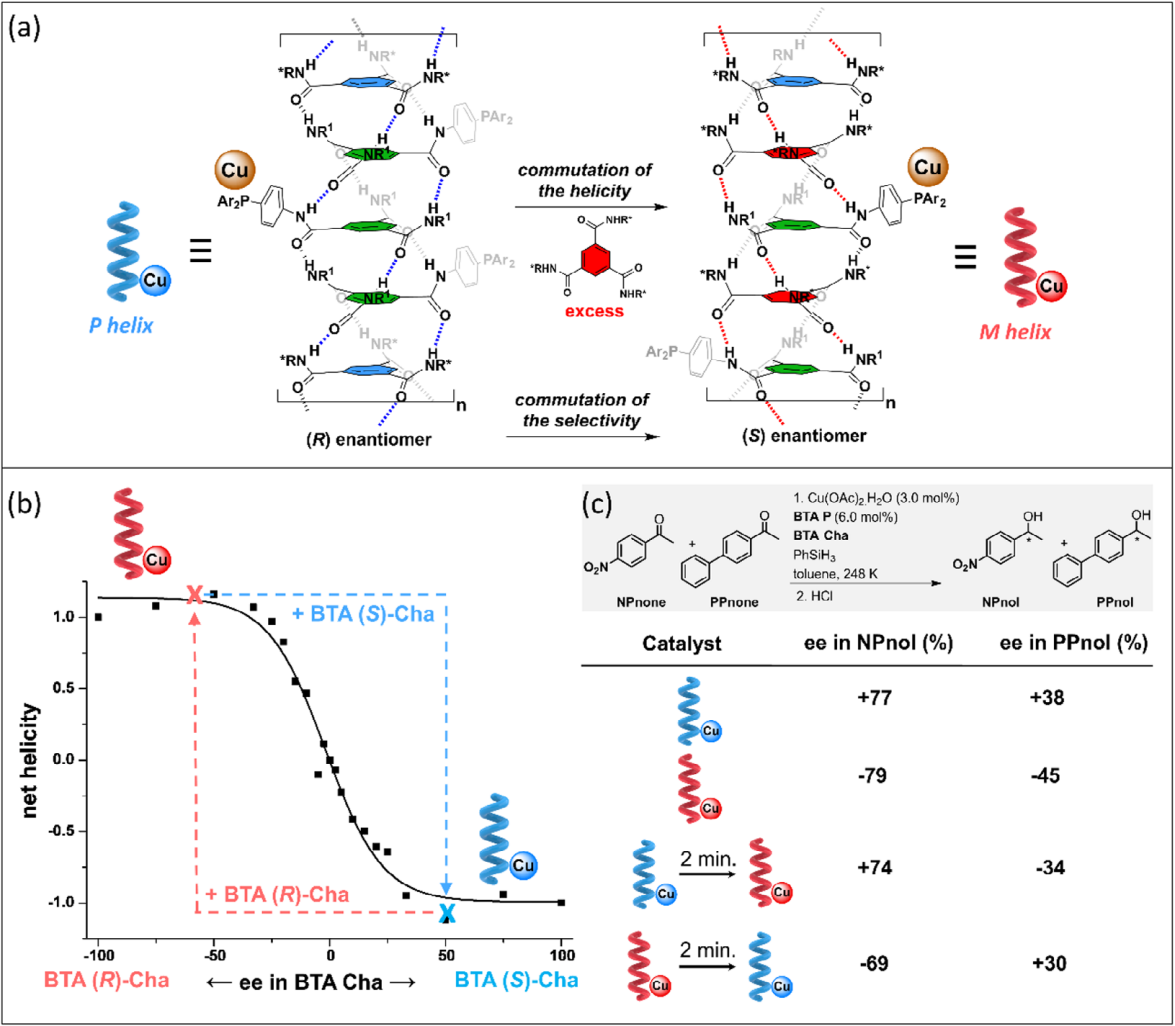
a) Schematic representation of the strategy used to switch the handedness and thus the selectivity of BTA‐based helical catalysts. b) Plot of the net helicity, that is, the optical purity of the supramolecular helices, as a function of the ee in “sergeants” for the mixtures composed of **BTA P** and both enantiomers of **BTA Cha**. c) Catalytic results obtained for the transformation of an equimolar mixture of **NPnone** and **PPnone** for BTA helical catalysts without and with commutation of the selectivity during the transformation.

A few catalysts built on a chiroptical switch can be predictably commuted between their two enantiomeric or pseudo‐enantiomeric states in the presence of the suitable stimulus.^[^ ^165^ ^]^ However, switching the selectivity in situ without compromising the catalytic process is very challenging. Combining achiral catalytically–active monomers and a nonequimolar (or scalemic) mixture of the “sergeant” enantiomers allowed us to design a new class of stereochemically switchable catalysts. Indeed, in this so‐callead diluted majority‐rule effect, the major enantiomer of the “sergeant” dictates its preferential helicity to both the minor enantiomer of the “sergeant” and the “soldier” (Figure 1). Adding the “sergeant” enantiomer initially in the minority in the system triggers an inversion of supramolecular terpolymer helicity, placing the catalyst in an opposite chiral environment (see a schematic representation of the concept in Figure 21a). The environments are fully commuted at the conditions that the system (i) is dynamic enough to integrate the added “sergeant” enantiomer, (ii) obeys the diluted majority principle, allowing homochiral helices to be obtained even though composed of a mixture of “sergeant” enantiomers.

In our initial system, consisting of **BTA P** and **BTA Cha** enantiomers, homochiral terpolymers were obtained for a scalemic mixture of “sergeants” of 33% ee or above (Figure 21b) which in turn yielded the hydrosilylation product with 88% of the optimal enantioselectivity. The selectivity of the supramolecular catalyst was switched in situ through sequential additions of the “sergeant” enantiomer (to switch the handedness). Starting with a scalemic mixture biased toward **BTA (*S*)‐Cha** (33% ee), the initial reaction yielded **(*R*)‐NPnol** in 53% ee. By adding **BTA (*R*)‐Cha**, the enantiomeric bias and handedness of the co‐assemblies were inverted. This procedure was repeated four times in total, confirming that the enantiomeric state can be switched in situ. The average selectivity of 54% ee per run was significantly higher compared to 21% ee in the case of a system displaying no diluted majority rule effect.^[^ ^151^ ^]^

The time required for the stereochemical switch was actually determined to be approximately 5 seconds, which is comparable to the reaction time of **NPnone**. Therefore, the stereochemical switch cannot be fully accomplished during the conversion of this substrate. However, the possibility to commute the selectivity during the hydrosilylation of a 1:1 mixture of **NPnone** and 1‐(4‐biphenylyl)‐ethanone (**PPnone**) was investigated; it was made possible by the lower reactivity of **PPnone**, allowing both substrates to be converted almost consecutively. Four experiments were conducted: two without the addition of the “sergeant” enantiomer, that is, the helicity and selectivity were not inverted during the reaction, and two with the addition of the “sergeant” enantiomer to trigger the commutation of the helicity and selectivity in situ. Reactions performed by single‐handed catalysts yielded **NPnol** and **PPnol** with the same preferred configurations, while reactions with supramolecular helical catalysts for which the handedness was switched after 2 minutes gave **NPnol** and **PPnol** with opposite preferred configurations. Thus, all four combinations of the two pairs of enantiomers were obtained, confirming the possibility to control the configuration of multiple stereogenic centers in situ with our designed BTA supramolecular helical catalysts.

**Figure 22:**
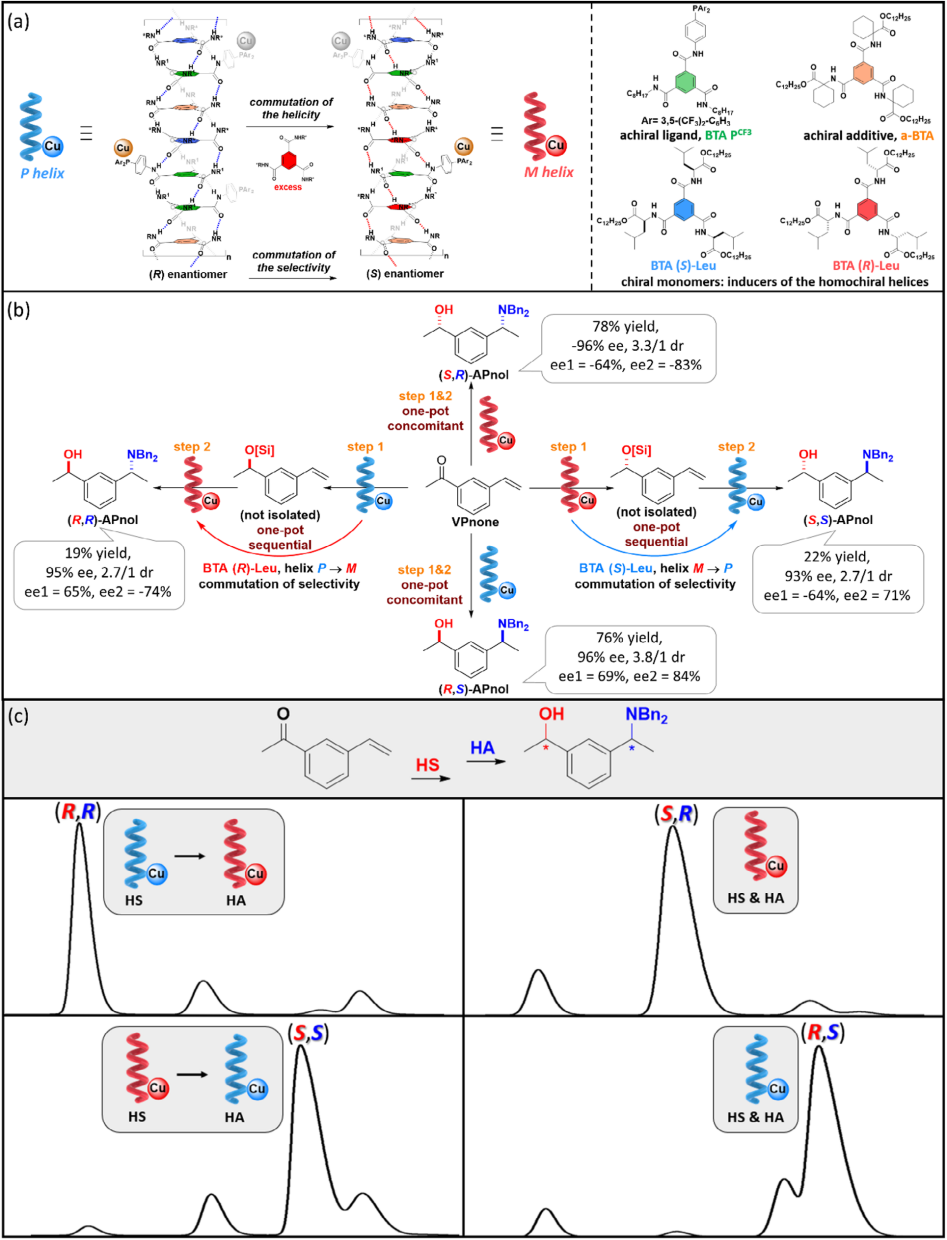
a) Schematic representation of the strategy used to switch the handedness of the BTA helical catalyst used in the cascade hydrosilylation/hydroamination reaction. Composition of the supramolecular ter‐ and tetrapolymer. b) Catalytic results obtained in the copper‐catalyzed hydrosilylation/hydroamination cascade transformation of **VPnone** with the BTA helical catalysts without and with commutation of the selectivity during the cascade transformation. Catalytic conditions: BTA monomers shown in a) P(3,5‐(CF_3_)_2_‐C_6_H_3_)_3_ dimethoxymethylsilane (DMMS) for both hydrosilylation and hydroamination, *O*‐pivaloyl‐*N*‐dibenzylhydroxylamine (amine transfer reagent) for hydroamination, toluene, 313 K. c) HPLC traces of **APnol**.

Benefiting from the aforementioned results, we envisaged the possibility to control the configuration of two stereogenic centers within the same molecular scaffold. 3‐vinylacetophenone (**VPnone**) was selected as a model substrate because it embeds ketone and vinyl functions, suitable for hydrosilylation and hydroamination reactions, respectively, two reactions that were efficiently promoted by our supramolecular helical catalysts (see Figures 16, 17, 18, 19, 20, 21).^[^ ^159^, ^161^ ^]^ BTA monomers that previously proved to be suitable for the copper‐catalyzed hydroamination of styrene were selected (Figure 22a). It included **BTA P^CF3^** as a “soldier”, **BTA Leu** enantiomers as “sergeants,” and **a‐BTA** as an achiral additive to improve the catalytic performance. Conditions without commutation of the catalyst handedness were first optimized. The expected product, 1‐[3‐(1‐dibenzylaminoethyl)]‐acetophenol, **APnol**, was obtained in better yield (76%) when P(3,5‐(CF_3_)_2_‐C_6_H_3_)_3_ was added as a secondary ligand. The first two stereoisomers of **APnol**, **(*S,R*)‐APnol** and **(*R,S*)‐APnol**, were thus obtained in high yield and enantioselectivity when **BTA (*R*)‐Leu** and **BTA (*S*)‐Leu** were used as the unique “sergeants” in the catalytic system (Figure 22b).

Subsequently, two additional experiments were conducted by adding the **BTA Leu** enantiomer after the hydrosilylation step, thus switching the handedness of the catalyst in between the hydrosilylation and hydroamination steps. Although **APnol** was obtained in low NMR yields (20–30%) under these sequential conditions, the expected stereoisomers became the dominant species. Enantiomeric excesses for each step have opposite signs as expected for reactions conducted with a catalyst displaying opposite intrinsic enantioselectivities. More specifically, the (*R,R*) and (*S,S*) stereoisomers of **APno**l were the major products when the handedness of the catalyst was switched from right to left and from left to right before the hydroamination step, respectively. By reducing the amount of **a‐BTA** to 5 mol% in the system, the (*R,R*) and (*S,S*) stereoisomers of **APnol** were obtained with a selectivity comparable to that of the concomitant process (Figure 22b).^[^ ^166^ ^]^ Chiral HPLC traces of the four reactions unambigously demonstrate the possibility to get the four stereosiomers of APnol with similar selectivities (Figure 22c). This work exemplified a new concept to achieve stereodivergency in which each stereoisomer can be obtained one‐pot thanks to a switchable asymmetric catalyst.

## 5 Summary and Outlook

Studies conducted in the last two decades unambiguously reveal that SPs and gels are not passive supports of catalytic sites engaged in asymmetric reactions but rather constitute an important lever to tune the catalytic performance and develop paradigms that are out of reach for conventional asymmetric catalysts. Examples reported to date are better classified according to the chiral or achiral nature of the monomer embedding the catalytic site since it allows to better disentangle the influence of the different chiral elements (molecular, supramolecular) on the enantioselectivity. Early examples of supramolecular gels and polymers revealed that chiral organocatalytic groups (mostly derived from proline) organized along 1D helical or tubular nanoassemblies performed better in model aldol reactions than their nonaggregated version in solution; advantages are mostly reflected in terms of rate and activity since enantioselectivity mostly stems from the molecular chirality located on the pyrrolidine unit. Subsequent studies with peptide amphiphile and bolaamphiphiles arranged into nanotubes or nanoribbons decorated with various metal centers in water clearly demonstrated that enantioselectivity may stem almost exclusively from the supramolecular chirality of the nanoobjects even though stereogenic centers are located in close proximity to the catalytically–active units. These systems constitute very modular platforms for constructing asymmetric catalysts. Key parameters for optimization include mixing functionalized and nonfunctionalized complementary monomers and tuning the monomer: metal ratio, while other conventional parameters such as concentration, solvent, temperature, and additives must be used cautiously as they may disrupt the supramolecular assemblies. An additional level of complexity was leveraged by the implementation of supramolecular helical polymers embedding achiral catalytically–active monomers. In those cases, chirality induction occurs by means of chiral monomers thanks to the “S&S” effect or through SMSB under specific conditions. Induction of chirality occurs at multiple scales allowing efficient asymmetric catalysis with sub‐catalytic amount of chiral inducers or even in the absence of chiral chemical species. Even though important parallel developments have been accomplished with dynamic helical covalent polymers,^[^ ^74^, ^80^, ^81^, ^82^, ^167^ ^]^ these two properties have been exclusively reported with catalytic supramolecular helical polymers. Finally, controlling the chirality by the nature of the major enantiomer of the chiral co‐monomer present in the catalytic SP through the diluted majority‐rule principle allows the development of a new class of switchable asymmetric catalysts, which have recently been implemented in a stereodivergent process.

Several challenges have to be alleviated for expanding the synthetic utility and applications of catalytic SPs and gels in asymmetric catalysis. Most of the examples reported to date are restricted to a few scaffolds, mainly peptides, porphyrin sulfonate, and benzene‐1,3,5‐tricarboxamide building blocks, which arrange into supramolecular structures driven mostly by hydrophobic and hydrogen‐bonding interactions, thereby limiting the nature of solvents suitable for their assembly to either water (or very polar solvents) or to very apolar solvents (toluene, alkanes), respectively. Tuning the chemical nature of the units is desired to strengthen the stability of helical SPs and gels, notably in the presence of moderately polar solvents or competitive reagents. Characterization of catalytic SPs by merging different analytical techniques^[^ ^168^ ^]^ is also key to better apprehend their structure, including the monomer sequence.^[^ ^169^ ^]^ Catalyst optimization by mixing monomers of different natures is a key advantage of catalytic SPs, which has not yet been fully exploited.^[^ ^170^ ^]^ In addition to this trial‐and‐error approach, which may be supported by high‐throughput screening, molecular simulations^[^ ^171^ ^]^ through computational and machine learning techniques should allow to get a more precise picture of the environment of the catalytic center, a key parameter to optimize the catalytic performance. The ability to switch between different stereochemical states of a single catalytic SP offers unprecedented control over reaction outcomes, which will be improved by better controlling the dynamic properties of the assemblies. Finally, expanding the scope of asymmetric reactions catalyzed by catalytic SPs beyond model transformations remains a significant challenge. Future research in this area would reveal the potential of catalytic SPs to trigger new catalytic processes and reactions.

## Conflict of Interest

There are no conflicts to declare.

## Data Availability

Data sharing is not applicable to this article as no new data were created or analyzed in this study.
